# Optimizing mobile app design for older adults: systematic review of age-friendly design

**DOI:** 10.1007/s40520-025-03157-7

**Published:** 2025-08-14

**Authors:** Elahe Amouzadeh, Iman Dianat, Javad Faradmal, Mohammad Babamiri

**Affiliations:** 1 Department of Ergonomics, Hamadan University of Medical Sciences, Hamadan, Iran; 2 Department of Occupational Health and Ergonomics, Tabriz University of Medical Sciences, Tabriz, Iran; 3 Modeling of Noncommunicable Diseases Research Center, Institute of Health Sciences and Technologies, Hamadan University of Medical Sciences, Hamadan, Iran and Department of Biostatistics, , School of Public Health,, Hamadan, Iran; 4 Department of Ergonomics, Research Center for Health Sciences, Institute of Health Sciences and Technologies, Hamadan University of Medical Sciences, Hamadan, Iran

**Keywords:** HCI, Age-friendly design, Usability, Elderly, Mobile applications

## Abstract

**Background:**

With the rapid growth of mobile technology, ensuring accessibility and usability for older adults has become a critical concern. This systematic review evaluates existing age-friendly mobile app design research, identifying key usability barriers and effective strategies for improving accessibility.

**Methods:**

This study reviews English-language research (Published between Jun. 2014 and Mar. 2025) on mobile applications for adults aged 60 + , focusing on user-centered design, usability testing, and age-friendly adaptations. It compares different levels of accessibility features and evaluates their impact on usability, satisfaction, and engagement across various settings. Studies were retrieved from four online databases (PubMed, Scopus, Web of Science, and IEEE Xplore).

**Results:**

This systematic review initially reviewed 1,556 records. From these, 132 articles met the inclusion criteria. The findings highlight several essential design elements, including simplified navigation, enlarged text and touch targets, voice interaction, and error-tolerant interfaces. Participatory design methods enhanced usability and satisfaction, demonstrating the importance of co-designing applications with older users. However, challenges like cognitive overload, lack of digital literacy, and accessibility barriers persist.

**Conclusion:**

The review emphasizes the need for future research on Artificial Intelligence-driven personalization, long-term usability studies, and culturally inclusive mobile applications. By integrating age-friendly design principles, developers can enhance digital inclusion, promote independence, and improve the overall well-being of older adults in an increasingly digital world.

**Supplementary Information:**

The online version contains supplementary material available at 10.1007/s40520-025-03157-7.

## Introduction

With the rapid development of mobile technology and its integration into daily life, mobile applications have become essential tools for communication, information access, and health management [1–4]. Given the prominent role of smartphones in everyday life, they must be designed to meet the diverse needs of users, especially groups with different abilities [1, 4]. However, older adults often face challenges in using mobile apps due to age-related declines in vision, motor skills, and cognitive function [1, 5]. With the aging population expected to double to over 2 billion by 2050 [6], designing mobile technology that accommodates this demographic is becoming increasingly important. designing mobile technology that accommodates this demographic is becoming increasingly important. While mobile apps offer older adults numerous benefits, such as health monitoring, social engagement, and convenient access to services, literature shows that they are often excluded from the design process[3–6]. This results in poor usability and low adoption rates [3–6]. Studies indicate that many mobile interfaces do not consider common age-related problems such as sensory limitations, reduced contrast sensitivity, or hearing impairments [3, 5]. Additionally, complex navigation systems and small touch targets can lead to frustration and abandonment of technology [5].

Experts in human–computer interaction, gerontology, and user experience design have proposed age-friendly design principles to address these barriers. Key recommendations include simplified navigation, larger fonts, voice-activated features, and error-tolerant interfaces [5]. However, there is no universally accepted framework for designing mobile apps optimized for older users. In this context, Mazuz and Biswas) emphasized the importance of co-designing apps with older users to understand their needs and preferences better, demonstrating how participatory design can help bridge usability gaps [7].

Given the lack of a standardized framework, it is essential to systematically examine the existing research to identify the most effective strategies for improving mobile app usability for older adults. Systematic reviews are crucial in consolidating findings from diverse studies and establishing clear, actionable guidelines for designers, developers, and policymakers. These guidelines can promote digital inclusion and enhance older adults' quality of life by fostering greater independence and participation in the digital world.

This systematic review aims to assess the current research on mobile app design for older adults, identify key challenges, and summarize effective strategies for improving usability and accessibility. Drawing from the latest literature, the findings will provide valuable insights for advancing the development of age-friendly mobile apps, ultimately promoting greater digital inclusion and improving well-being among older populations.

## Methods

### Study Design

This systematic review followed the Preferred Reporting Items for Systematic Reviews and Meta-Analyses (PRISMA) guidelines to answer the following question: “How can user experience for older adults in mobile applications be improved by integrating age-friendly features?”.

### Search Strategy

A comprehensive search was conducted using the following electronic databases: PubMed, Scopus, Web of Science, and IEEE Xplore. Additional searches were performed in conference proceedings and relevant grey literature sources to reduce publication bias. Searches were limited to English-language articles published between 2014 and 2025. The search terms and Boolean operators used included:

Search Terms:

The search terms and Boolean operators used included:

- **Search Terms**:
  o ("Design, User-Centered" OR "Designs, User-Centered" OR "User Centered Design" OR "User-Centered Designs" OR "Usability Testing" OR "Testing, Usability")
  o AND ("elderly" OR "older adult" OR "aged" OR "aging")
  o AND ("Application" OR "App" OR "Apps" OR "Mobile App" OR "Smartphone App").

To identify additional relevant publications, we manually screened the reference lists of selected studies. The complete search strategy is available in Supplementary Materials.

## Eligibility Criteria

The study's eligibility criteria were established in advance using the PICO (population, intervention, comparisons, outcomes) framework, and the content validity was reviewed and confirmed by the research team members (EA, MB, JF):

*Population:* Studies investigating mobile applications targeting older adults aged 60 years and above.

*Intervention:* Studies focusing on user-centered design, usability testing, or age-friendly technological adaptations.

*Comparison:* Mobile applications with varying age-friendly features or those without age-friendly features.

*Outcomes:* Improved user experience metrics such as accessibility, ease of use, satisfaction, engagement, and overall usability.

*Time/language*: English-language articles published between January 2014 and January 2025.

*Setting*: There were no limitations based on the type of settings.

Studies that focused on technologies or applications unrelated to mobile apps, research that included general populations without specific segmentation for older adults, non-peer-reviewed studies, editorials, and opinion pieces were excluded.

## Study Selection

Titles and abstracts of identified records were screened for relevance using Rayyan. Articles not meeting inclusion criteria were excluded. Full texts of potentially relevant studies were retrieved and assessed for eligibility based on predefined criteria by two independent reviewers (EA and MB). Any disagreements between reviewers were resolved through discussion, and, if necessary, a third reviewer (ID) was consulted. Studies that met all inclusion criteria after full-text review were included in the final analysis.

### Quality assessment

The methodological quality of included studies was assessed using the Mixed Methods Appraisal Tool (MMAT, version 2018). The results of the quality assessment are provided in Supplementary Table 1. Studies with high risk of bias (21.2%, scoring 1/5 or 2/5) were noted but included in the narrative synthesis.

## Data Extraction

- Data extraction was carried out using a data extraction form, which included: study characteristics: author(s), publication year, country, study design, sample size, age range, and gender distribution.
- Technology and design features: name of the mobile app, Artificial Intelligence (AI)/Technology used, UX evaluation tools, technology experience of participants, key age-friendly features.
- User experience and outcomes: accessibility, usability metrics, engagement, satisfaction, and perceived barriers.
- Challenges and future directions: barriers faced by older adults, the impact of design features, and recommendations for future studies.

## Data Synthesis

The findings were synthesized qualitatively to identify recurring themes, common approaches, and potential gaps in integrating age-friendly features in mobile applications for older adults. Quantitative synthesis (e.g., meta-analysis) was not performed due to anticipated study design and outcome heterogeneity.

### Results

The systematic review identified 1556 articles through the initial database search. After deduplications were removed, title and abstract screening, 154 included full-text reviews. Ultimately, 132 studies met the inclusion criteria and were included in the analysis (see PRISMA flow diagram in Appendix 1).

### Quality assessment

The methodological quality of the 132 included studies was assessed using the Mixed Methods Appraisal Tool (MMAT). MMAT scores ranged from 1/5 to 5/5, with 51 studies (38.6%) achieving a perfect score of 5/5 due to the robustness of their study designs and the strong integration of qualitative and quantitative data. Mixed-methods studies (39.4% of total) generally exhibited higher quality, reflecting effective integration of data collection and analysis methods. Descriptive studies with unspecified sampling strategies (20.5%) scored lower, often receiving 1/5 or 2/5 due to a lack of details on sampling and representativeness. Common weaknesses included unreported response rates in descriptive studies and a lack of reporting on the influence of researchers in qualitative studies. The variability in methodological quality should be taken into account when interpreting the findings of this review.

### Characteristics of included studies

**4**.4% of the studies were conducted in China. The included studies comprised experimental studies (18.2%), mixed-methods research (39.4%), usability testing (27.3%), and intervention studies (15.1%). Sample sizes varied across studies, ranging from N = 10 to N = 200, with participants aged 60 to 99 years. The gender distribution was balanced in some studies, while others had a higher representation of either male or female participants. A summary of the included studies is presented in Table 1.

**Table 1:** Summary of Included Studies on Age-Friendly Mobile App Design

S. no.	Authors	Sample size and age rang	Gender distribution	Study type	Prototype name	AI/technology used	UX evaluation tools	Technology experience	Intervention (age-friendly features)	| Age-friendly design features	Main challenges for older users	| Impact of design features	Suggestions for future research	country
1.	Al-khomsan, et al. [96]	N = 30 60-75 ys	50% male, 50% female	experimental	Not specified	Not specified	Morae evaluation software	Not specified	Not specified	Not specified	Not specified	Improved satisfaction, reduced errors, faster task completion, higher ease of use scores	Not specified	Saudi Arabia
2.	Ahmed, A., et al. [20]	N = 100 55–75 + ys	64% male, 37% female	Mixed-method	Not specified	iOS (iPhone 3GS, 4S) and Android (Samsung Galaxy 2, Note 2)	Novice, intermediate, and experienced older adults	Usability testing, surveys, and post-test questionnaires	Proposed design with pictorial gestures to reduce cognitive load	Gesture-based interfaces tailored to older users; focus on icon-based navigation	Issues with small screen size, text readability, functional complexity, and error tolerance	Enhanced usability; reduced cognitive stress; increased user satisfaction and efficiency	Not specified	**Pakistan**
3.	Al-Shaher, and Abdul-wahed (2020) [97]	Not specified	Not specified	Design and implementation research	Not specified	Android development using Java programming, SQLite, and Eclipse IDE	Usability testing	Not specified	Medication adherence tracking, heart rate monitoring, online doctor appointment booking	User-centered design, Material Design, easy navigation, health monitoring, and appointment scheduling	Difficulty with medication adherence, health data tracking, and navigating small mobile interfaces	Improved usability for health management, better patient-doctor interaction, time-saving features for appointment booking		**Iraq**
4.	Ali, Z., et al. (2024) [22]	N = 45 primary care providers Age range = Not specified		Mixed-methods (qualitative and quantitative)	CareMOBI	mHealth app (CareMOBI) for dementia care, no specific AI mentioned	Interviews, surveys, usability testing		Dementia-focused features like reminders, communication aids, and tracking tools	Simple navigation, user-friendly interface, health monitoring for dementia	Cognitive decline, app usability, health data accessibility	Increased usability among providers, better dementia care coordination	Further exploration of mobile health interventions, user-centered design, and testing with broader healthcare providers.	**Singapore**
5.	Aljedaani, and Alnanih (2023) [98]	N = 20 Age range = Not specified	Not specified, but participants were older adults (including those with Alzheimer's)	Usability testing, experimental	Spaced Retrieval Therapy Mobile Application (SRT App)	Mobile application for spaced retrieval therapy (SRT)	Usability testing, post-task questionnaires	Participants included both healthy elderly adults and those with Alzheimer's, all with varying levels of familiarity with mobile applications	wo interfaces were tested: a default interface for elderly people and an adapted interface for those with cognitive impairments	Simplified interface for general elderly users and an adapted interface for Alzheimer’s patients, focusing on ease of navigation and cognitive support	Difficulty in completing tasks, especially for Alzheimer’s patients, and complexity in using the mobile app	Alzheimer’s patients performed better and faster with the adapted interface, while healthy elderly participants preferred the default interface	Further optimization of mobile interfaces for elderly users, especially those with cognitive impairments, and additional usability testing across different stages of Alzheimer's disease​	**Saudi Arabia**,
6.	Alkhomsan, M. N., et al. (2023) [1]	N = 20 Age range = Not specified	Not specified	Usability evaluation, qualitative interviews, heuristic evaluation, cognitive walkthrough	m-government application with two interfaces (default and adapted	Not specified; m-government mobile application designed with cultural and cognitive considerations	Usability testing, cognitive walkthrough, heuristics evaluation, interviews with elderly users	Elderly users with varying levels of technology experience, including cognitive impairments	Two interfaces designed for elderly users, one standard and one adapted for cognitive impairments	Simplified navigation, cognitive load reduction, culturally sensitive design for Saudi elderly users	Cognitive impairments, physical limitations, unfamiliarity with technology, challenges in using m-government applications	Adapted interface better suited for Alzheimer's patients, simpler default interface preferred by healthy elderly users	further research on the usability of mobile applications for elderly users, considering cognitive and cultural factors, and testing across different government services	Saudi Arabia
7.	Alsaqer, M. and S. Chatterjee (2017) [23]	Not specified	Not specified	Usability testing, persuasive mobile intervention development	AdBo (Adherence Booster)Exercise app	Mobile app, health tracking, memory games, reminders	Usability testing, feedback from elderly users, memory testing, performance tracking	Varies among elderly participants; designed to address cognitive decline and limited technological familiarity	Push notifications, video instructions, memory games, daily performance reports	Simple interface, motivational videos, easy-to-understand instructions, progress tracking, memory improvement games	Cognitive decline, difficulty adhering to exercises, physical limitations, memory loss	ncreased adherence to physical exercises, improved cognitive tracking, motivation through peer stories and feedback	Further development and evaluation with real-user feedback, assessing the long-term impact on adherence and cognitive improvemen	USA
8.	Alvarez, et al. (2017) [24]	Not specified	Mostly female caregivers, as is typical in caregiving populations (from studies in similar contexts)	ntervention study, usability testing, and mobile app evaluation	apps such as STAV (Support to Family Caregivers) and others like Caring Village, Lotsa Helping Hands	Mobile applications designed for caregiver coordination, stress management, and physical health monitoring	User feedback, usability testing, interviews, data analysis of caregiver app usage	Caregivers of elderly patients, varying levels of experience with technology	Features like reminders, caregiver coordination, stress management tools (e.g., breathing exercises), and easy communication channels	Simple user interface, easy navigation, stress management tools, and progress tracking	Cognitive decline, physical strain, lack of technological literacy, and difficulty in coordinating caregiving tasks	Improved caregiving coordination, reduction in caregiver stress, better health management, and improved communication among caregivers	Further evaluation of specific needs of elderly caregivers, the role of community support through apps, and long-term impact of mobile interventions	uk
9.	Anastasiadou and Lanitis (2022) [25]	Not specified	Not specified	Mixed-methods	VR Application	Not specified	Usability Testing, Surveys	Social interaction, virtual community engagement		Not specified	Social isolation, user experience complexity	Reduced social isolation, increased engagement	Exploration of virtual reality interventions for elder well-being	USA
10.	Ariza-Vega, et al. (2024) [26]	Not specified	Mixed	Qualitative	mHealth intervention	Not specified	Interviews, Focus groups	Moderate	Physical therapy, social support	User-centered design, caregiver collaboration	Complexity, usability	Enhanced rehabilitation, emotional support	Scalability, longitudinal studies	UK
11.	Arruda, et al. (2018) [99]	N = Not specified Age range = 65	Mixed	Framework	Mobile device framework	Not specified	Usability Testing, Surveys	Varies	Simplified UI, training modules	Accessibility, ease of use	Increased device adoption, user satisfaction	Expansion to other technologies, usability refinement	Further research on cross-cultural usability aspects and testing across diverse aging populations	Brazil
12.	Azam, W. A. I. W. A., et al. (2021) [83]	N = Not specified Age range = 70	Mixed	User preference study	eText interface	Not specified	Focus groups, Surveys	High	Adjustable text settings, voice options	Enhanced readability, high contrast	Improved reading experience, independence	Assistive technology integration, accessibility	Further research on AI-assisted interfaces and personalization for elderly users	Malaysia
13.	Bhattacharyya, O., et al. (2019) [82]	N = Not specified Age range = 60	Mixed	Human-centered design	Digital health advisor	Personalized recommendations, analytics	Persona development, Testing	Varied	Care management, real-time health monitoring	Customizable interfaces, intuitive navigation	Enhanced patient engagement, holistic care	Enhanced usability, improved adherence to health programs, increased digital engagement	Further exploration of AI-driven personalization and long-term user engagement strategies	Canada
14.	Bhayana, R., et al. (2020) [100]	N = Not specified Age range = 72	Mixed	Application	Sahayak application	Not specified	Surveys, Observations	Moderate	Community engagement, physical activity tracking	Social connectivity, health monitoring	Increased social interactions, improved health outcomes	Expanding social features, integrating AI		Colombia
15.	Boccardi, A., et al. (2022) [101]	10 participants; aged 60-83 years	Mixed	Mixed Methods	Wheelchair Maintenance App	Not specified	Surveys, Observations	Moderate	Simplified navigation, assistive features	Accessibility, ease of use	Enhanced maintenance, user satisfaction	Improved usability and acceptance among older users	Further usability testing post-revision, larger sample size studies	USA
16.	Calderón-Gómez, et al. [102]	32 PD patients (5, 50–74) + 25 healthy controls	18 M, 14F (PD); 13 M, 12F (Control)	Cross-sectional observational study	Telemonitoring System	Machine Learning	Predictive modeling, alerts	High	Real-time monitoring, predictive analytics	Data privacy, system reliability	Early detection of diseases, improved outcomes	Expansion to more diseases, patient-centric feedback	Longitudinal studies on auditory deficits in PD	spain
17.	Calyam, et al. (2017) [78]	N = Not specified Age range = 70	Mixed	Living Lab	ElderCare Living Lab	Sensor Technology	Health Assessment, Therapy	Moderate	User-focused design and feedback	Multi-sensory feedback, real-time data analysis	Integration challenges, privacy concerns	Comprehensive health monitoring, personalized care. Proactive health monitoring and remote therapy	Expansion to other health domains, AI integration	usa
18.	Carrisa, et al. (2022) [28]	N = Not specified Age range = 60	Mixed	Case Study	Mobile Chat Room Interface	Not specified	Automated Testing, Usability Testing	High		Simplified navigation, clear communication	Age-related impairments, usability issues	Improved interaction, reduced errors	Continuous testing, feedback loops	**Indonesia**
19.	Casciaro, S., et al. (2020) [29]	N = 12 Age range = 65	Mixed	Smart Device User study and preliminary evaluation	Smart Pill Dispenser	Medication Management	Observation, interviews, usability testing	Moderate	Medication reminders, monitoring intake, caregiver alerts	Simple display, light and sound notifications, companion mobile app	Forgetfulness, health monitoring Difficulty interacting with the device, forgetting medication, need for a simpler UI	Increased adherence, safety featuresImproved medication adherence, reduced medication errors	Expansion to telemedicine, AI insights Enhancing device design, long-term evaluation with larger groups	Italy
20.	Chang, A. S. Y., et al. (2022) [73]	N = Not specified Age range = 70	Mixed	Mobile App	Meal BentoGo! APPDelivery App	GPS positioning, delivery confirmation, emergency notification, batch mode non-real-time data transfer	User-Centered Design (UCD) methodology, observation, standardized interviews	Moderate Delivery workers previously relied on pen-and-paper methods	App designed to improve meal delivery efficiency for rural seniors living alone	Easy access, real-time updates GPS tracking, meal information display, emergency contact notification, offline batch mode data transfer	Food insecurity, inefficiency due to paper-based methods, poor mobile data transmission	Improved meal delivery, social inclusion	Expansion to other rural services, IoT integration Evaluating workflow efficiency improvement, assessing delivery worker satisfaction	Taiwan
21.													Iterative design, regular updates	korea
22.	Charissis, et al. (2024) [31]	N = Not specified Age range = 65	Mixed	XR Design	Virtual Rehabilitation	XR Technology	Exergaming, Physical Therapy	High		Immersive experiences, adaptive feedback	Limited mobility, cognitive strain Ensuring safety, maintaining motivation, considering social factors	Increased engagement, physical improvement Improved rehabilitation efficiency, increased motivation, ease of use at home	Expansion to other therapeutic areas, AI feedback Developing design recommendations for XR-based exergames for motor rehabilitation	Norway
23.	Chen, and Gao (2021) [32]	Not specified	Not specified	Not specified	Not specified	Image features, pedestrian gait rerecognition	Not specified	High	Community activity space update	Environmental perception	Behavioral needs, usability	Positive role in self-management	Symbiosis and sustainable development	china
24.	Chen, et al. (2022) [33]	2,270 Android apps (not focused on age range)	Not specified	Empirical Investigation	Android apps accessibility xbot	Accessibility testing Automated accessibility testing, static program analysis	Usability metrics	Not specified	Not specified	Accessibility features Lack of proper UI labeling, missing accessibility features	Usability barriers Poor accessibility in Android apps	Enhanced accessibility Identified 86,767 accessibility issues affecting usability	Encouraging developers to integrate accessibility in design	**China**
25.	Chien (2024) [35]	18 patients with COPD; age range not specified	Usability Study	Mobile app for COPD	Health tracking, exercise guidance	Not specified	High	Exercise adherence	Home-based pulmonary rehabilitation monitoring	Personalized feedback, simplicity	Breathability, symptom tracking Difficulties with touch screens and scroll menus	Improved health outcomes High satisfaction; no critical errors; patients took longer to complete tasks	Incorporate intuitive design aids like auditory support and visual health progress indicators	Taiwan
26.	Chirayus, and Nanthaamornphong (2020) [36]	Not specified	Preliminary Study	Cognitive mobile design	Accessibility features, simplicity	Not specified	High	Cognitive support	Compilation and analysis of mobile design guidelines for elderly users	Large fonts, simple navigation Cognitive-based improvements in mobile UI/UX	Understanding complexity Design flaws affecting cognitive abilities of elderly users	Enhanced usability Improved usability and accessibility	Empirical studies with elderly participants to refine guidelines	**Thailand**
27.	Choi, J., et al. (2021) [74]	15 older adults (Phase I: 5, Phase II: 10); Age range not specified	Not specified	Descriptive exploratory study using qualitative methods	Tab-CBI (Tablet-Based Cognitive Behavioral Intervention)	Tablet-based application incorporating cognitive behavioral therapy (CBT) principles	Cognitive walkthrough with a think-aloud technique	Not specified	Walking support Multimedia CBT-based educational modules, videoconferencing, individualized goal setting, and electronic data submission	Simplified navigation, step Guidance Intuitive interface, video recordings, and videoconferencing for enhanced user engagement	Fatigue management, physical strain Difficulty holding the tablet during videoconferencing	Enhanced physical activity support Increased motivation for walking and improved confidence in managing fatigue	Further engagement with cognitive tasks Refine Tab-CBI based on user feedback and conduct pilot studies on larger populations	United States
28.	Cordasco, et al. (2014) [103]	Not specified	Not specified	Usability evaluation	vassist system prototype vAssist	Voice recognition, Natural language processing	Usability testing	Moderate	Speech recognition issues, comprehension difficulties	Enhanced voice navigation, accessibility y Hands-free operation for users with limited mobility	Improving conversational interfaces	Expanding functionality for varied tasks Improved accessibility for individuals with physical impairments	Further studies to validate findings in real-life contexts and long-term user adaptation	**Italy**
29.	Cornet, et al. (2020) [104]	Not specified	Qualitative research focusing on design and evaluation	Design and Evaluation	Mobile health app	Not specified	Heuristic evaluation, User interviews	High	Accessibility issues, cognitive load Mobile health app for older adults with heart failure, focusing on user-centered design	Improved symptom tracking, personalized alerts	Future enhancement of user engagement Practical challenges in implementing UCD for mHealth in older adults with heart failure	Expanding health data integration, reducing cognitive overload Aims to improve usability and engagement for older adults with heart failure	Further research to address UCD challenges and involve stakeholders in the design process	**Netherlands**
30.	Correia, G. S., et al. (2024) [87]	Not specified	Methodological study with an applied quantitative approach	Usability Study	ROBOVID app	Mobile application for health education	Heuristic evaluation, User testing	High	Accessibility issues, information overload Educational tool for COVID-19 awareness	Enhanced educational content, ease of navigation	Further improvements in user engagement	Tailoring content for different age groups Complies with usability principles, demonstrating efficiency, effectiveness, and user satisfaction	Future studies involving a broader demographic to enhance applicability	Brazil
31.	Craioveanu, and Marcu (2023) [105]	Not specified	Not specified	Usability Study	Adaptive Music App	Real-time location tracking, music generation	Usability testing, subjective feedback	High	Personalized auditory experiences, real-time adaptation	Cognitive support for elderly travelers	Enhanced comfort during travel	Further exploration of diverse auditory experiences for varying demographics	Further testing for long-term effects	Romania
32.	Cristiano, et al. (2018) [106]	N = 30 Age range = 65	Not specified	Validation Study	Roto-translating seat	Not specified	Biomechanical analysis, interviews, checklists	Objective biomechanical measures, subjective feedback	Adaptive seat movement to reduce trunk and knee motion during car ingress and egress	Reduced trunk and knee range of motions, lower muscle fatigue Reduces the range of motion, muscle fatigue, and fall risk	Risk of falls during ingress/egress Difficulty in performing movements due to age-related decline	Improved mobility and safety Effectively assists with entry and exit, reduces fatigue and fall risk	Further testing of system in real-world settings	italy
33.	Curiel, et al. (2014) [107]	Not specified	Not specified	Comparative Study	HiSozial Platform	Person-oriented interfaces, service encapsulation	Usability testing, comparative analysis	Low to moderate	Unified access to multiple communication services, simplified interface	Enhanced accessibility through service encapsulation and personalization	Digital divide, interaction difficulties Difficulty navigating complex interfaces and accessing various communication services	Improved communication access Increased user satisfaction, enhanced usage of communication services among elderly users	Further refinement of personalized interfaces	**Germany**
34.	Czuber, N. K., et al. (2024) [10]	6 focus groups, 30 usability testing Age range = 65-85	Mixed (Majority Female)	Mixed	Fall Prevention Exercise App	Behavior change strategies	Usability testing, focus groups, expert meetings	Moderate	Exercise prescriptions tailored to individual needs, integration with primary care workflows	Exercise prescription, evidence-based content	Lack of adherence, complex interface	High user satisfaction, tailored exercise recommendations	Further research to explore visual clarity	United States
35.	Darmawan, et al. (2023) [108]	N = 155 Age range = Not specified	Mixed	Mixed	PeduliLindungi Mobile App	Privacy, responsiveness	Offline & online surveys, AMOS 24.0	Moderate		Content, billing, availability	Data leaks, slow service	Improved privacy, efficiency	Further enhancements for responsiveness	Indonesia.
36.	De Melo, et al. (2016) [37]	N = 21 Age range = 65 +	Mixed	Case study	ElderNote	N/A	Usability testing, performance	Moderate	Mobile application technology	Reminder features, accessibilitcy User-centered design focusing on elderly users' needs	Cognitive decline, usability issues Interaction differences, trust issues, fears, functional complexity, and motivation aspects	High usability and accessibility	Future studies on broader demographics	brazil
37.	Diewald, S., et al. (2015) [79]	N = Not specified Age range = 65 +	Mixed	User-centered design	Mobile fitness app	N/A	User testing, focus groups	Moderate	Designed to assist older adults in engaging with physical fitness activities	Fitness features, accessibility	Interaction differences, trust issues	High sensitivity to user feedback	Enhanced user testing methods	germany
38.	Doménech, et al. (2023) [109]	N = 56 Age range = 65 +	Mixed	Observational study	LONG-REMI	AI-supported therapy	VAS scale, PANAS scale	Participants engaged with the app over four weekly sessions	Intangible cultural heritage AI-driven reminiscence therapy based on intangible cultural heritage aimed at improving emotional well-being	Managing cognitive impairment and engagement User-centered design tailored to older adults' cognitive abilities	Increased positive emotions	Further refining personalized therapy features High usability and satisfaction scores; significant improvement in positive affect scores (28.86 to 36.70)	Further studies with larger samples to confirm findings and refine the application	Spain and Portugal
39.	Dos Santos, M. M. T., et al. (2016) [11]	N = 35 Age range = Not specified	Qualitative research	Prototype-based study	ANNI	Customizable interface	Interview, prototype testing	Participants' experiences with tablet applications	Personalized health-related applications designed to meet preferences of older adults and caregivers	Interactive, social functionalities	Complexity of interface and social features	Enhanced interaction and usability	Further enhancing caregiver-specific features	**Brazil**
40.	Držanič, et al. (2019) [38]	N = 50 Age range = Varies	Mixed	Usability study	TeleStiki	Telecare application	Think-Aloud, SUS, UEQ	Varies	Safety, reduced caregiver burden, increased independence		Perceived complexity, learning curve	Enhanced usability, increased user confidence	Future integration of AI-driven features	**Slovenia**
41.	Dworschak, C., et al. (2024) [63]	N = 12 Age range = > = 65	Mixed	Development study	7-module CBT intervention	Internet-based CBT	Semi-structured interviews, usability testing	Low to moderate	Individualization, interactivity	Content customization, interactivity, age-specific support	Technical barriers, missing content (philosophy/theology) Technical barriers, lack of content on philosophy/theology, and role of descendants/relatives	High acceptance, reduced loneliness, personalized approach	More content on relevant topics, further exploration of technical barriers	Germany
42.	Eicher and Ursprung (2024) [110]	N = Not specified Age range = Varies	Mixed	Development study	HEROES App	Remote recruitment, video applications	User-centered approach, group discussions	Varies	Trust-building, inclusive design, data protection	Recruitment speed and cost reduction, trustworthiness in caregivers	Enhanced trust, privacy, and user engagement	Continued testing and adaptation for diverse needs	Further studies on usability, expanding app features to more sectors	**Switzerland**
43.	Eun, et al. (2022) [111]	N = 37 Age range = 60-80	Mixed	Pilot Study	AI-Based Cognitive Exercise Game	Difficulty level adjustment, web-based mobile application	Basic technology usage	Cognitive training	Playful elements, difficulty adaptation, accessibility	User engagement, cognitive improvement, immersive experience	Further exploration of AI tools, advanced visualization	Improved cognitive performance	Long-term studies on effectiveness, AI personalization	South Korea
44.	Ferre, et al. (2017) [64]	28 participants (7, 21)	Mixed	Iterative Development Study	Ultrasonic sensor-based gait speed measurement device	Mobile interface, ultrasonic sensors	Usability testing, technical validation	Basic to moderate technology use	Self-assessment of physical performance	User-friendly mobile interface, remote monitoring	Reduced mobility, frailty, cognitive decline	Early detection of frailty, improved monitoring	Further research on the effect of gait speed on cognitive and frailty outcomes	United States (Multisite study)
45.	Forbes, et al. (2024) [112]	N = 30 Age range = 75 +	Mixed	Qualitative Study	Mobile Health Apps, Telehealth tools	mHealth apps, telehealth	Thematic analysis, qualitative research	Varied technology use, some unfamiliarity	Technology adoption, sociocultural design Simplified navigation, culturally relevant content, clear instructions	Accessibility, user interface usability, emotional interaction	Low perceived ease of use and usefulness due to lack of sociocultural elements	Incorporation of cognitive and cultural frameworks improves usability Low perceived ease of use and usefulness, lack of engagement due to design flaws	incorporating cognitive and cultural elements in mHealth design, understanding elderly technology	United States
46.	Frechette, M., et al. (2022) [113]	N = 10 Age range = Not specified	Mixed	Observational Study	Steady-Wheels App Smartphone-based fall risk assessment app	Mobile Health App	Systematic Usability Scale, semistructured interviews	Varied experience with mobile health apps	Fall risk assessment, remote usability	Guided navigation, intuitive design, error recovery Large buttons, high contrast, simple instructions	Easy-to-use, color-coded risk reporting Difficulty using smartphone for elderly wheelchair users, physical limitations	Improvement in design led to better usability scores	Further testing with larger, diverse samples, and advanced features for accessibility	United States
47.	Frogren, J., et al. (2018) [114]	N = 19 Age range = 66-93	Mixed	Feasibility Study, Usability Testing	SMART4MD Model App	mHealth application	Task-based usability tests, structured interviews	Varied exposure to similar technology	Health-oriented customization	Self-esteem and ability affected by less exposure to technology	Pre-cautions needed for usability evaluation	Positive feedback on the simplified and intuitive design, improved usability for cognitive impairments	Further testing with larger sample sizes, additional features for diverse cognitive impairments	Sweden
48.	Fuglerud, K. S., et al. (2018) [115]	Not specified	N/A	Design and Testing	APPETITT Tablet App Healthy eating inspiration app	Tablet application	User feedback, awareness creation	Varied technology experience	Healthy eating inspiration, nutritional guidance	Home-dwelling older adults organizing meals	Low engagement, nutritional awareness Cognitive decline, limited tech literacy, memory loss	Pictures of meals, quality nutrition info	Further testing for usability and broader adoption	Norway
49.	García-Crespo, A., et al. (2020) [75]	N = 9 Age range = Not specified	N/A	Usability Study	GoAll Mobile App	Mobile application, Braille display	Usability survey, application server data	Varied technological interaction	Direct content access through CCs Direct access to broadcasted content through assistive technology	Deafblind individuals Audio feedback, tactile feedback, simplified interface	Limited Braille speed, CC constraints	Greater autonomy for users Increased autonomy, improved accessibility to television content	Further refinement of accessibility features	Spain
50.	Giraldo, F. D., et al. (2015) [39]	N = 40 Age range = Not specified	N/A	Case Study	Android Prototype	Mobile application	Usability testing, interviews	Limited technological experience	Telecare system to improve elderly care in a senior facility	Simple interface, health monitoring features	Low SES, limited access to technology, cognitive impairments	Increased access to healthcare services, ease of use for caregivers	Focus on improving user support features	Colombia
51.	Griffin et al. (2019) [90]	N = 50 Age range = 50-85	Mixed gender	Case Study	CRC screening app	Virtual Human Technology	Usability testing, surveys, interviews	Varied technological experience	Health education, screening	User feedback loops, interactive content	Age-related cognitive barriers, usability concerns	Enhanced app credibility, usability, effectiveness	Focus on more accessible technology features	USA
52.	Ha, S., et al. (2023] [116]	N = 27 Age range = Not specified	Mixed gender	Usability Study	Daily Healthcare 2.0	IoT devices, PGHD integration	Heuristic evaluations, usability testing	Varied technological experience	Care coordination, tailored educational content	Home menu complexity, task-focused navigation	Increased eHealth literacy, caregiver engagement	Development of more accessible interfaces		South Korea
53.	Hakobyan, L., et al. (2014) [76]	Not specified	Mixed gender	Participatory Design	SMART	Mobile application	PICTIVE participatory design	Varied technological experience	Self-monitoring, ability-reactive design	Collaborative design, user involvement	Vision impairment, ease of navigation	Increased engagement in design, user-centered features	Tailored support for visual impairments	**Canada**
54.	Hammour, G., et al. (2024) [117]	N = 17 Age range = 65-83	Mixed gender	Transfer Learning	Ear-EEG	Machine learning model	LightGBM, feature-based transfer learning	Varied technological experience	Non-invasive, continuous sleep monitoring	Remote monitoring, non-invasiveness	Data accuracy, model calibration	Enhanced sleep stage classification	Further exploration into elderly-specific EEG processing	**Lebanon**
55.	Hanghøj, S., et al. (2020) [41]	N = 20 Age range = 16-29	Mixed gender	Co-creation, Usability Study	Kræftværket	Mobile phone app	Think-aloud, Thematic analysis	Varied technological experience	Symptom tracking, information search	Gender-specific differences in app use	Relevant tracking features	Notifications, post counting	Further refinement and testing with a broader AYA population	**Denmark**
56.	Happe, et al. (2022) [42]	N = 49 Age range ≥ 65	49% female	Usability Study, Iterative Testing	Nutrition and mobility e-coach	Tablet-based e-coach	System Usability Scale (SUS)	Varied technology experience	Nutrition, mobility, behavior change	Focus on education, comprehension	Navigation and comprehension challenges	Consistent chart types, explanatory notes	Further studies on long-term usability	**Germany**
57.	Harte, R., et al. (2018) [43]	N = 22 Age range = 65-85	Not explicitly stated	Usability Study, Training Intervention	Fall prediction system	Smartphone-based system	Satisfaction rating, time, cues, errors	Limited technology proficiency	Smartphone app, fall prediction	Basic smartphone training	Navigation, syncing challenges	Improved task completion with training	Further research on long-term impact	**Ireland**
58.	Harte, R., et al. (2017) [118]	Not specified	Data not explicitly provided	Usability Study, Human-Centered Design (HCD)	Fall risk detection system app	Smartphone interface	NASA Task Load Index (NASA-TLX)	Limited technology proficiency	Human-Centered Design	Clear feedback, low cognitive demands	Recurring themes in feedback: clear, relevant feedback and minimal anxiety-inducing elements	Improved elimination of usability issues through HCD phases	Further refinement of HCD methodologies for diverse older adult populations	**Ireland**
59.	Heiney, S. P., et al. (2020) [62]	N = 12 Age range = 51-69	Data not explicitly provided	Feasibility Study, Usability Testing	Healthy Heart app	Smartphone interface	Health Related Quality of Life Scale (HQOL14), Self-Care of Heart Failure Index (SCHFI)	Limited technology proficiency, low health literacy	Human-Centered Design	Text-based one-way messages, journaling, graphical data display, customized feedback	Low health literacy and limited smartphone experience were significant challenges	Clinically relevant changes observed in self-care maintenance, management, and confidence	Explore effectiveness for a larger and more diverse sample of older adults	United States
60.	Huang, J., et al. (2024) [119]	N = Not specified Age range = 45-70 years	Virtual Square Dancing	Middle-aged and elderly individuals	Smartphone-based app for virtual square dancing	Collaboration with square dance communities	Human-Centered Design, Co-design	UN SDG 3 (Good Health and Well-Being), UN SDG 11 (Sustainable Cities and Communities)	Social engagement, physical activity	Motivating physical activity & social interaction	App successfully increases physical and social well-being	Technology fosters more inclusive urban communities for the elderly		**China**
61.	Huwa, J., et al. (2023) [80]	N = 100 Age range = Not specified	Mixed (Majority female)	Usability & Acceptability	2wT Intervention	SMS-based digital intervention	Mixed-methods usability & acceptability	Low to moderate technology experience	Reminders and motivational messages	Visit reminders, appointment changes	Privacy concerns, literacy issues	High user satisfaction (95%)	Further research on accessibility for non-mobile users	South Africa
62.	Iqbal, S., et al. (2020) [120]	Not specified	Mixed	Proof of Concept	Fingerprint-based digital wallet	Usability questionnaires	Low to moderate technology experience	Secure, easy-to-use payment mechanism	Simplified fingerprint verification, Bluetooth-based billing	Authentication security, physical dexterity	High satisfaction and ease of use Security concerns, unfamiliarity with technology	ncreased accessibility, reduced need for passwords	Further testing with a larger elderly population	Likely Pakistan
63.	Ismail, N. A., et al. (2021) [121]	N = 10 Age range = Not specified	Mixed	Usability Testing	Touch-based & multimodal interaction	Usability testing data	Moderate to low technology experience	Simplified swiping gestures replacing buttons	Swiping gestures, simplified interfaces	Task completion time, satisfaction	Elderly preferred swiping gestures	Further testing with larger sample size and other smart home applications		Malaysia
64.	Jakkaew and Hongthong (2017) [122]	Not specified	Not specified	Usability Study	Persona, Prototype, Scenarios, Heuristic Evaluation	Not specified	Limited to moderate technology experience	Usability-focused mobile UI for elderly	Simple navigation, large fonts, limited features	Understanding and usability challenges	Prototype used to refine usability Limited familiarity with technology	Improved usability and accessibility	Further research on feature preferences and usability testing	Likely Thailand
65.	Jiang, et al. (2024) [123]	Not specified	Not specified	Factor Analysis	Age-Friendly Mobile News App	Not specified	Usability testing, interviews	Varied	Improving accessibility and usability for elderly users	Larger fonts, high contrast, simplified navigation	Cognitive and visual impairments, unfamiliarity with digital news platforms	Enhanced readability and ease of use	Future work on AI-driven personalization and adaptive interfaces	Likely China
66.	Kalyani, N. L., et al. (2022) [44]	Not specified	Not specified	Development Study	Flip60 LIP60-AR	Augmented Reality (AR)	Not specified	Limited tech-savviness	Yoga and exercise activities, AR models, recommendation system	Realistic augmented reality models, video recommendations	Difficulty in interacting with AR features Lack of familiarity with AR, difficulty in adapting to new technology	Enhances exercise engagement and user experience	Further testing and refinement of AR features	India
67.	Kangeswaran, V., et al. (2021) [68]	Not specified	Not specified	Assistive technology and accessibility study	Bilingual Audio-Based Online Shopping App	Text-to-Speech (TTS), Voice Recognition	Usability testing, user feedback	Limited accessibility Varied	Audio-based product descriptions, voice navigation, text-to-speech functionality	Audio guides, voice commands, text-to-speech for descriptions	Difficulty navigating traditional e-commerce apps	Improved ease of use and accessibility	Expansion to more languages, integration with AI-based personalization	India
68.	Kim, H., et al. (2020)[6]	N = 20 Age range = Not specified	Not specified	Development Study	Active Senior AppSocial Relationship App	Not specified (likely AI-based recommendations)	Usability and utility testing	Personalized leisure activity information, social interaction features	Strengthening social relationships among older adults	Simple UI, social engagement features, notifications	Digital literacy, usability concerns	Increased social interaction and engagement	More personalization, AI-driven social suggestions	South Korea
69.	Kim, S. B., et al. (2024) [124]	N = 100 Age range = Not specified	Not specified	Usability Study	Healthcare App	Sound feedback, font size, preparatory phase	Performance Matrix, System Usability Scale (SUS)	Limited familiarity with technology	Assistance, font size increase, sound feedback, preparatory phase	Improved usability	Facilitates cognitive support for elderly	Future research on broader application development tailored for elderly users		South Korea
70.	Klimova, B. and L. Sanda (2021) [65]	N = 13 Age range = 55 +	Not specified	Pilot Study	Educational App	Visual interface, easy navigation	Usability Testing	Limited technology experience	Technical (visual interface, navigation) & pedagogical aspects	User-friendly features, clear instructions, instructional manualSimple interface, engaging content, visual aids	Cognitive decline, lack of digital literacy	Improved cognitive engagement, user satisfaction	Further empirical studies needed for educational apps tailored to seniors	Czech Republic
71.	A User-centred Design Approach for Mobile-Government Systems for the Elderly[125]	Not specified	Not specified	Mixed Methods	m-Government Prototype	IGUAN Framework	Usability Testing, Qualitative and Quantitative Analysis	Limited technology experience	Usability and security-focused features	User-driven approach focusing on usability, perceived security	High acceptance rates due to enhanced usability and security Limited digital literacy, navigation challenges	Enhanced accessibility, better engagement	Further studies on larger diverse populations needed	**Hungary**
72.	Kunaratana-Angkul, Y., et al. (2020) [69]	Not specified	Not specified	Usability Evaluation	Not specified review	Visual field simulation tools for designing visual interfaces	Usability testing, interviews	Varied	Use of parameters suitable for individuals with color blindness and control of light/shadow in design	Parameters tailored for individuals with color blindness; optimized light and shadow control	Vision issues such as color blindness and the need for proper light/shadow control in user interfaces	Improved app usability for elderly users with low vision through optimized visual interface design	Development of personalized interfaces for diverse visual impairments	Taiwan and Thailand
73.	Liu, Y. C., et al. (2019)	N = 105 (Young adults: 70, Older adults: 35) Age range: 18-29 (young), 55-73 (older)	Not specified	Randomized Controlled Trial	Self-chosen tab app and autonomous exhaustive list app	Not specified	Not specified	Not specified	Customized dietary recording apps designed for both young and older adults	User-centered design; combinatorial concept for dietary recording	Potential neglect in selecting food attributes by older users in the self-chosen tab app	High accuracy (> 98%) in both apps; self-chosen tab app more time-efficient	Further development of diversified dietary recording prototypes; consider user interaction improvements	**Taiwan**
74.	Liu, Z. Y. and X. R. Yu (2024) [9]	Not specified	Not specified	Participatory Design Study	Not specified	Not specified	Heuristic Evaluation, Usability Testing	Not specified	Customization for elderly users with diabetes, simplified navigation, and user-friendly design	Customization for elderly users with diabetes, simplified navigation, and user-friendly design	Complexity in app navigation, ease of use issues for elderly users	Improved usability for elderly users managing Type 2 Diabetes through age-friendly design	Further research in diabetes-focused apps	China
75.	Margaritini, A., et al. (2022)[126]	Not specified	Not specified	Study protocol	Not specified	Not specified	Not specified	Not specified	Use of social robots for nursing support	Adaptive communication and task management for homecare	Challenges in physical assistance and emotional support	Improved support for nurses and patients	Further investigation on long-term impacts and integration	
76.	Mehra, S., et al. (2019)[70]	N = 15 Age range = 69-99	Not specified	Usability Evaluation	Not specified	Visual feedback systems	Mixed-methods	Varies (novice users)	Goal setting, tailoring, progress tracking, remote feedback	Tailored interface for low-vision, simplified tasks	Vision issues, difficulty in complex task execution	Effective basic task performance, overall satisfaction	Long-term usability through follow-ups	Netherlands
77.	Merilampi, S., et al. (2017)[127]	28 elderly patients & 6 experts	Not specified	Participatory Design Study	SugarShift	None specified	Interviews, observations	Not specified	Cognitive self-rehabilitation games for elderly users	Culturally tailored content; cognitive stimulation Simplified navigation, logical operation, reduced cognitive load	Cultural adaptation, usability across diverse contexts Issues with information display, system feedback, and user interaction	Improved usability, better information processing, increased retention	Simplify data entry, improve readability, enhance feedback mechanisms	china
78.	Molnar, T. (2015)[67]	N = 75 Age range = Not specified	Not specified	Usability Evaluation	AusweisApp of the electronic ID card	Not specified	Iterative prototyping	Varies (elderly users and students)	Development of the IGUAN guideline for usability improvement in e-government systems	Catalog of criteria addressing elderly user requirements; iterative prototyping for usability improvement	Lack of familiarity with interactive applications; need for tailored e-government systems	Increased acceptance and motivation for using e-government systems among elderly users	Further testing of the IGUAN guideline in diverse applications and contexts	Italy
79.	Mondellini, M., et al. (2018) [45]	Not specified	Not specified	Usability Pilot Study	Virtual Supermarket	Virtual Reality (VR)	Self-reports, objective performance measurements	Healthy young adults (pilot test for elderlies)	Immersive environment for cognitive training (supermarket simulation)	Sense of presence, enjoyment, and clear interaction design	Cybersickness, unclear product visualization, and interaction modalities	Good usability and promising cognitive engagement	Explore improvements in accessibility and navigation	**Italy**
80.	Morris, L. D., et al. (2024) [12]	Not specified	Not specified	Case study on microwave ovens	Not specified	None specified	Coding scheme for interaction analysis	Not specified	Evaluation of usability for domestic appliances, focusing on older adults	Information processing activities, clear interfaces, and workflow efficiency Simple interfaces, intuitive controls, visual cues	Problem-solving barriers, interface complexity, unclear instructions Difficulty in appliance interaction, cognitive overload	Improved usability of interfaces for independent living	Further exploration on adapting features for cognitive decline	**United Kingdom**
81.	Nair, A. B., et al. (2022) [13]	N = 15 Age range = Not specified	Not specified	Usability Evaluation	Simple Android Launcher	Flutter (App Development)	Feedback-based usability testing	Minimal experience with smartphones	Simplified access to essential smartphone features like contacts and emergency calling	Simplified UI, emergency calling, medicine checklist	Struggles with smartphone usage due to cognitive decline, complexity in features	Enhanced adoption of smartphones, reduced digital gap among elderly	Broader demographic testing, long-term usability analysis	**India**
82.	The Experience of the Elderly in Using Applications: Case Study of a Banking Application[128]	Not specified	Not specified	Heuristic Evaluation Pilot Study	Immersive Virtual Supermarket	Virtual Reality (VR)	Heuristic-based usability evaluation	Limited experience with technology	Adaptations based on usability and accessibility heuristics	Improved navigation, accessibility features for elderly users	Frustration, insecurity, disorientation in using technology	Enhanced engagement and cognitive stimulation	Expanding usability testing to elderly users, optimizing VR interaction	italy
83.	Park, G. E., et al. (2024) [129]	18 older adults (mean age 74 ± 3 years), 8 experts	90% women	Development and usability study	HAHA2022	Wearable device integration	Korean version of Mobile Application Rating Scale	Likely moderate experience with apps	Digital health coaching, integrated tracking with wearables	Age-friendly UI - Integration with wearables for tracking - Simplified navigation	Difficulty in managing multiple chronic conditions - Limited technology experience	- Usability problems linked to interface complexity - Poor instructional material design leads to interruptions	Further effectiveness testing - Wider trials with different populations	South Korea
84.	Petrovčič, A., et al. (2019) [46]	15 elderly volunteers	Not specified	Developmental/Experimental	MDPQ Android Elderly Launcher	Objective smartphone skills	Confirmatory factor analysis	Varies based on experience	Mobile device proficiency, mobile apps	Customizable and accessible features	Difficulty in learning, adapting to interfaces, lack of personalization Complexity of standard smartphone UIs - Small fonts/icons - Difficult navigation	Validity and reliability of MDPQ for older users, improvements in smartphone utilization	Further adaptation of MDPQ to address age-specific needs and barriers to usage	**Slovenia**
85.	Pinto, M. and P. Marques (2017) [130]	N = 60 Age range = Not specified	Mixed (not specified)	Proof of concept, usability study	OneCare Spiro	Wireless medical devices, spirometry	Satisfaction survey using Likert scale	COPD patients with physical limitations and technology illiteracy	Remote monitoring, symptom tracking, pre-diagnosis features	Usability issues, technology literacy	High satisfaction (6.65/7), technical feasibility	Further user testing for broader adoption and long-term use		**Portugal**
86.	Porcel Gálvez, A. M., et al. (2024) [131]	Piloted in six countries	Not specified	Platform development and usability testing	TEC-MED Platform	Clinical decision support system, standardized nursing taxonomies (NANDA-I, NOC, NIC)	Usability testing in 6 languages	Focus on healthcare professionals, collaboration among academics and IT developers	Multilingual platform to improve nursing care for older adults Standardized nursing language in assessment module	Multilingual interface (English, Spanish, French, Italian, Greek, Arabic), evidence-based practice support	Language support in Arabic, platform functionality (offline use, mobile app limitations)	mproved care through standardized communication, clinical decision support, accessibility in multiple languages	urther testing to evaluate effectiveness, cross-cultural validation, and improvements in Arabic language support	Piloted in six Mediterranean countries
87.	Puebla, C., et al. (2022) [132]	N = 22 Age range = 60 +	Not specified	Design-thinking, prototype development, pilot testing	Language Learning App Prototype	Features for social and collaborative learning, peer matching	Pilot testing in individual evaluation sessions	Focused on older adults, likely mixed levels	Social and collaborative language learning tool for older adults	Promotes face-to-face interaction, active lifestyle, learner-centered experience, hobby-based group matching	Overlooked needs in existing language-learning apps for older adults Challenges in technology adoption, usability for seniors	Improved social interaction, engagement, and learning opportunities in a collaborative setting	Further research to refine app functionality, evaluate long-term impact on language acquisition for older adults	Mediterranean countries (six countries involved)
88.	Putri et al., 2019 [86]	Not specified	Not specified	User-Centered Design (UCD) approach, design pattern modeling	Not named	Focused on design patterns for recurring UI/UX problems	Not specified	Designed for elderly with special characteristics	Mobile application for scheduling daily activities for seniors	Templates to address design problems; tailored for elderly needs; tested through iterative processes	Issues with activity scheduling applications due to lack of elderly-friendly interfaces	Simplified scheduling, improved accessibility, and enhanced activity organization for elderly users	Explore adaptability of design patterns across different application domains and demographics	**Indonesia**
89.	Quesada, L., et al. (2024) [49]	Not specified	Not specified	Multi-case study involving six applications	Multiple prototypes (career guidance system, senior games, etc.)	Intelligent personal assistants (Alexa, Google Assistant, Siri, Cortana)	Standardized questionnaires, customized surveys, expert evaluations	Not specified	Voice applications tailored to diverse contexts, including games for seniors and educational fairs	Simplified voice interactions, task-specific functionality, inclusive design for elderly users	Challenges in natural language processing, user adaptability, and interface learning curves Difficulty in understanding and using AI assistants	mproved accessibility and interaction	Further AI adaptation for elderly users	**Costa Rica**
90.	Quintana, M., et al. (2020) [50]	N = 19 Age range = Not specified	Not specified	Feasibility and usability testing	SMART4MD Tablet App	Tablet-based health application	Task-based usability tests, satisfaction surveys	Limited or no experience with eHealth	Reminder and monitoring functionalities for individuals with mild dementia, caregiver support	Simplified UI, large icons, clear text, intuitive navigation, and task-based testing	Cognitive limitations, difficulty in adapting to new technology, caregiver burden	Enhanced user satisfaction (81% users satisfied), usability insights contributed to refinement of app design	Explore longitudinal effects of app use on patient and caregiver outcomes, assess scalability to other cognitive conditions	**pain, Sweden, Belgium, and the Czech Republic**.
91.	Radhakrishnan, K., et al. (2016) [51]	= 40, Age range: Older adults with heart failure (exact range not specified)	Not specified	Feasibility and usability testing	HF Self-Management Game	Serious gaming technology	Observations, usability surveys, Atlanta Heart Failure Knowledge Test, Self-Care for Heart Failure Index (pre/post-test)	Varied, some with low digital literac	Heart failure self-management education through gamified learning	Engaging gameplay, personalized feedback, simple UI, clear instructions	Cognitive challenges, limited experience with technology	Improved self-care behaviors, increased engagement in health management	Longitudinal studies to assess long-term effects, adaptation for other chronic conditions	USA
92.	Raghavendra, K., et al. (2024) [52]	N = 100 Age range = 60-75	Not specified	Usability testing and psychometric study	Not specified	Adaptive UI/UX based on user behavior patterns, voice-task models	Observation, psychometric analysis, task completion tests	Limited technology exposure	Customizable UI/UX designs for banking, finance, and cab booking apps tailored to older adults’ needs	Adaptive interfaces, task simplification, voice-based interaction models	Cognitive, sensory, and physical challenges when using smart devices	Increased user engagement, reduced technology anxiety, and improved task efficiency	Investigate long-term adaptability, apply the adaptive UI approach to diverse app categories, and test with broader demographics	**India**
93.	Ran, D., et al. (2024) [133]	Not specified	Not specified	Empirical investigation and guideline design	Not specified	Not specified	Not specified	Likely varied among participants	Guidelines to adapt popular apps to address elderly users' specific needs	Simplified UI, larger text/icons, streamlined workflows	Difficulty with navigation, small font size, lack of intuitive design	Enhanced usability and accessibility for older adults	Development of specific tools to automate UI adaptation for elderly users; explore applicability across app categories	**China**
94.	Rangel, P., et al. (2019) [14]	Not specified	Not specified	Development of SAR prototype	Personal Assistance Humanoid Robot	Open-source technologies	Object recognition, motion monitoring, human gesture emulation	Basic experience with robotics	Assistance with personal care, vitals, motion monitoring	Simple, intuitive interface, humanoid design, social interaction capabilities	Technological complexity, physical limitations	Enhances social interaction, reduces loneliness, promotes independence	Further evaluation in real-world settings, scalability of open-source platform	United States
95.	Rasche, et al. (2015) [53]	N = 15 Age range = 60 +	Not specified	Empirical Study with 4-week duration	Activity Tracker	Motion sensors, step measurement	Post Study System Usability Questionnaire (PSSUQ) for usability evaluation	Limited experience with wearable devices	Designed to track physical activity, promote healthy lifestyle	Simple interface, step tracking, motivational elements	Limited experience with technology, mental effort in understanding the device	Activity trackers accepted, motivating, and have suitable usability for elderly	Need to further refine design to reduce mental effort, explore additional features for elderly needs	Germany
96.	Rath and Chandna [134]	Not specified		Conceptual/Feasibility Study	Voice Assistive Technology for Health Monitoring	Voice Assistants (AI)		-	Limited experience with technology, limited knowledge of health monitoring tools	Voice commands, hands-free interaction, integration with health monitoring	Cognitive limitations, physical disabilities, difficulty adapting to new technology	Enhances health monitoring through voice commands, hands-free operation	mprove accuracy of voice recognition, integrate more health-related features, test with larger user groups	india
97.	Reading Turchioe, et al. (2020) [71]	N = 56, Age Range: 65-95	63% male	Cross-sectional feasibility study	Inclusively designed app	Mobile health technology	Health Technology Usability Survey (Perceived Ease-of-Use, Usefulness)	Moderate technology experience	Inclusively designed mobile application for PROMIS measures	Inclusive design, ease of navigation, intuitive interface	Limited accessibility features for those with significant visual or physical impairments	High perceived usability regardless of age	Emphasize visual and physical accessibility for older adults with impairments	United States
98.	Riaz, et al. (2021) [15]	Not specified	N/A	Qualitative analysis	Mobile phone interfaces	N/A	Usability testing, mental health and physical condition considerations	Low to moderate technology experience	Inclusive design, simplified interfaces, touch sensitivity	Simplified navigation, larger fonts, fewer buttons	Difficulty in interacting with complex interfaces, small text, low contrast, lack of familiarity	Improved usability through modifications in interface design	Further studies on cultural and regional differences in smartphone usability among older adults	Pakistan
99.	Rodríguez-Dueñas, et al. (2021) [66]	Not specified	N/A	Usability pilot study	Mobile care app	N/A	Usability testing, user-centered design methodology	Varies by user	Simplified navigation, remote monitoring, caregiver communication	Larger fonts, voice commands, easy-to-use interface	Lack of familiarity with mobile technology, accessibility issues	Improved accessibility and ease of use	Further usability testing and integration of AI-driven features	**Colombia**
100.	Rosman, et al. (2023) [16]	Not specified	N/A	Usability testing	BruHealth	N/A	Heuristic evaluation, user-centered design model	Varies by user	Simplified navigation, voice commands, larger text sizes, intuitive design	Clear menu structure, high-contrast visuals, voice guidance	Technology unfamiliarity, small interface elements, complex navigation	Improved usability for elderly, alignment with age-related impairments	Future studies exploring diverse mobile applications for older adults	**Singapore**
101.	Saari, and Hynninen (2021) [54]	Not specified	N/A	Usability testing	Touchscreen puzzle game	N/A	Usability tests, observations, interviews	Low, as they were new to gaming	Animated tutorials, pop-up messages, simplified navigation	Animated tutorials, pop-ups, voice guidance	Difficulty starting the game, need for instructions, different mental models	Enhanced usability with tutorials and feedback Improved usability awareness	Investigate long-term user adaptation	**Finland**
102.	Schaaf, et al. [55]	N = 8 Age range = Not specified	N/A	Qualitative study	COMTRAC-HIV app	N/A	Focus groups, thinking-aloud tests (TA test), video/audio recordings	N/A	Privacy considerations, medication tracking, image uploads	User-interface clarity, medication reminders, health tracking	Privacy concerns, non-intuitive controls, illogical button placement	Enhanced care, user satisfaction despite usability issues	Refinement of functionality, focus on clinical and privacy needs	**Germany**
103.	Shi-Ning and Yuanqing (2022) [47]	Not specified	N/A	Qualitative study	Housekeeping APP	N/A	Semi-structured interviews, factor analysis, questionnaire analysis	N/A	Social interaction, cognitive fitness, service expectations	Social interaction, cognitive fitness, intended use, service expectations	Cognitive limitations, social isolation	Enhanced social interaction, usability guidance	Further exploration of user preferences and technological integration	**China**
104.	Shore, et al. (2020) [135]	N = 11 Age range = Not specified	N/A	Pilot study	Soft Lower Limb Exoskeleton	N/A	Exoscore design evaluation tool, iterative design process	N/A	Perceptions of technology, user feedback, design optimization	Concept design, usability, comfort, independence	Comfort, usability, cognitive accessibility	Iterative improvements, testing with larger sample size Increased technology acceptance	Further testing with more elderly participants	**Ireland**
105.	Siangpipop, et al. (2023) [17]	N = 10 Age range = Not specified	N/A	User-Centered Design	Alzheimer's App	N/A	Design thinking, usability testing, scenario tests	Limited experience	Simple UI design, minimal screen elements to avoid confusion	User-friendly, movement tracking, caregiver features	High learning curve, confusion with complex interfaces	Improved usability, caregiver-focused features	Further testing with larger sample sizes and diverse user groups	**Thailand**
106.	Sien, et al. (2024) [18]	N = 1 Age range = Not specified	N/A	User-Centered Design, Co-Design	Symptom Management App	N/A	Design thinking, thematic analysis	Limited experience	Tracking functions, clear display, medication reminders	Symptom tracking, clear UI, medication management	Confusion with multi-functional features	Supports symptom management, medication tracking	Further large-scale testing and longitudinal studies	**Canada**
107.	Sifan, et al. (2021) [77]	Not specified	N/A	User-Centered Design, Android Development	Intelligent Assistant App	N/A	Voice recognition, touch-based interaction	Limited smartphone experience	Personalized customization, information assistance, health reminders	Touch, vision, voice interaction	Difficulty with voice commands and touch inputs	Provides personalized information, health alerts	Further usability testing and feature refinement	**China**
108.	Silva, et al. (2015) [56]	N = 10 evaluator, Age range = Not specified	N/A	Heuristic Evaluation	Nike + , Run keeper	Nielsen's Heuristic Evaluation	Positive assessment of heuristics and usability issues	Limited experience	Clarity, simplicity, navigation, feedback, accessibility	Comprehensive application, user-friendly	Identified gaps in UI consistency, error handling, and user feedback	Enhancing error correction, improving usability testing	Further refinement of heuristics and broader testing	**the United States** or **Europe**,
109.	Sinabell and Ammenwerth (2024) [136]	Not specified	Mixed (not specified)	Triangulation Study	Agile Usability	Iterative expert interviews, exploratory case study	Agile feedback mechanisms	Not specified	Prospective user involvement, iterative development for older adults	Addressing age-related impairments in usability evaluation	Implementing agile methods effectively	Further development of eHealth usability methods	Further evaluation of agile methodologies in eHealth	germany
110.	Smith-Turchyn, et al. (2017) [137]	N = 10 Age average = 68.4	Not specified	Usability Study	TAPESTRY-CM	Not specified	Cognitive interviewing, semi-structured interviews	Moderate/low	Self-management focus, user-centered design	Content clarity, layout simplicity, ease of use	Positive feedback for content, need for layout improvements	Improving content and layout for broader usability	Further usability studies with larger sample sizes	canada
111.	Sobnath, et al. (2016) [138]	Not specified	Not specified	Usability Study	WELCOME	Wearable Sensing and Smart Cloud Computing	Questionnaires, interviews	Moderate	Self-management, remote monitoring, program integration	Ease of use, program engagement, remote functionality	Positive feedback for usability and acceptability	Evaluating long-term impact and user engagement	Further study on long-term usability	United kingdom
112.	Sobrinho, et al. (2024) [4]	N = 20 Age range ≥ 60	Mixed (Gender-specific)	Usability Study	Health App	-	SAM, SUS	Moderate/High	Health promotion, user engagement, personalization	Font size, navigation, visual feedback, personalization, social interaction	Installation, web-based search difficulties	Increased satisfaction and usability; health device integration	Exploring long-term usage, accessibility, and scalability	brazil
113.	Son, et al. (2023) [139]	N = 43 Age median = 74	Mixed (Gender-specific)	Usability Study	Online FC	-	SUS	Moderate	Self-assessment of sarcopenia, questionnaire for diet, physical, and social behaviors	Online screening, self-assessment, usability testing	Age, educational level, ICT proficiency	Marginally high acceptability, reliable for continuous frailty screening	Enhancing accessibility, improving navigation and personalization	**Japan**
114.	Son and Kim (2023) [57]	N = 26 Age median = 62	20 men	Mixed-Methods Study	WithUs	eHealth	Usability Testing	Moderate	Self-care behaviours, eHealth literacy, disease knowledge	Mobile app, routine self-care, disease monitoring	Adaptation challenges, app adoption	Improved self-care behaviours, eHealth literacy, disease knowledge	Enhancing adaptability, reducing barriers to technology use	**South Korea**
115.	Teh, et al. (2024) [140]	N = Not specified Age range = 60 +	Varied	Qualitative Study	AGATHA	Gamified platform	Interviews, usability testing	Varies (low to moderate)	Interactive features, decision-support, digital literacy	Comprehensive content, user-friendly design	Need for peer interaction, personalization, improved UX	Enhancing digital literacy, fostering an inclusive digital environment	Larger-scale trials in other populations, long-term engagement evaluation	**Malaysia**
116.	Tonga, et al. (2021) [141]	N = 27 Age range = Not specified	Varied	Mixed Methods	RA Hand Exercise App	None	USE, SUS, Semi-structured interviews	Varied (low to moderate)	Self-monitoring, exercise diary, behavioral change	Usability, satisfaction, ease of use	Motivation and adherence, ease of use	Improving interactive features, long-term adherence	Long-term study on physical health impact	**Turkey**
117.	Toyota, et al. (2014) [58]	Not specified	Not specified	Usability Testing	Demonstration App	None	Subjective evaluation	Limited smartphone experience	Hands-on real video demonstrations, self-learning	Basic smartphone operation, confidence building	Understanding challenges in smartphone use	Self-learning, confidence-building Increased familiarity with smartphones and digital learning	Further usability improvements, focus on learning retention	**Japan**
118.	Tran-Nguyen, et al. (2022) [142]	N = 27 Age median = 70	Not specified	User-Centered Design	Low-Fidelity/Mid-Fidelity/High-Fidelity Prototypes	None	Surveys, Workshops	Limited smartphone experience	Support resources, diary entries, educational materials	User interface, ease of use, educational content, medical accuracy	Usability, feedback on user experience, suggestions for improvements	Further evaluation of clinical outcomes and long-term effects	Further studies on long-term pain management effectiveness, adaptation to other health conditions	canada
119.	Tu, et al. (2023) [143]	N = 30 Age range = Not specified	Not specified	Usability Testing	Spring Rain Doctor App	None	Web-based questionnaire	Limited smartphone experience	Visual checklist with usability questions	Simplified visual interface, task-specific usability focus	Accessibility for older rural users	Identified areas for optimization	Explore digital literacy programs, improve network access	China
120.	Ubam, et al. (2021) [144]	N = 36 Age range = 55 +	Not specified	Usability Testing	None	None	Questionnaire	Limited smartphone experience	Fast loading time, intuitive navigation, simple task flow	Simplified interface, focus on essential features	Preference for essential features like quick transactions	Improved usability, secure transactions	Test with larger senior citizen group, focus on security features	**Malaysia**
121.	Wahab, et al. (2021) [145]	Not specified	Not specified	Quantitative Survey	FinTech applications	None	Questionnaire	Varied technology experience	Simplified interface, clear instructions, security features	Simplified financial tools, easy navigation	Focus on usability for the elderly and security	Increased trust and satisfaction, secure financial transactions	Focus on improving trust and security features	**pakistan**
122.	Wang, et al. (2024) [146]	N = 148 Age range = various	Not specified	Randomized Crossover	3D-DST Mobile App-Based Clinical Decision Support System for Delirium	None	Usability Questionnaire	Varied technology experience	Simplified navigation, built-in reminders, error reduction	Simplified delirium assessment process, reduction in human error	Complex information, technical issues	Improved clinical decision-making	Further clinical testing with diverse populations	**China**
123.	Wang, et al. (2022) [147]	Not specified	Not specified	Usability Study	Railroad 12306 Love APP	None	Comparative Usability Analysis	Varies technology experience	Simplified interface, reduced error rates, improved satisfaction	Enhanced design for older adults, easier ticket booking	Usability and efficiency for older adults	Future usability testing and optimization for aging users	Conduct more studies with diverse groups, test under different conditions	china
124.	Xiong, et al. (2019) [72]	Not specified	Mixed gender	Qualitative/Quantitative	Online Learning Mobile App	None	Observation, Questionnaire, Interview	Varies technology experience	ARCS motivation model to enhance online learning engagement	Improved user experience and learning efficiency	Difficulty with navigation, motivation to learn	Increased engagement and satisfaction	Further usability testing and model refinement	chaina
125.	Xu and Wen (2024) [148]	Not specified	Mixed gender	Mixed Methods	CH32V307VCT6 Robot	Deep Learning, MQTT	None	Embedded technology	Real-time remote monitoring with AI-based fall detection	Flexibility and efficiency in remote monitoring	Wireless communication and accurate fall detection	Expansion of features and integration with home care systems	Further usability testing and model refinement	chaina
126.	Yee et al. (2024) [149]	Not specified	Mixed gender	Mixed Methods	FitBot	ChatGPT, Personalized AI	None	Limited AI usage	Personalized healthcare and self-care for chronic disease management	Enhanced self-care through tailored fitness tracking	Overcoming comprehension barriers with AI responses	Integration of more health indicators and interactive features Enhanced fitness engagement, health monitoring	Further testing with larger sample sizes	tiwan
127.	Yeo, et al. (2015) [59]	Not specified	Mixed gender	Mixed Methods	Hearing Aid App	Speech-to-Text, Visual Conversion	None	Limited technology exposure	Real-time and non-real-time speech-to-text conversion	Noninvasive, easy-to-use, inexpensive app	Difficulty in real-time conversion and understanding text visually	Enabling independent and fulfilling lifestyles	Further development of real-time and multi-language support	**Singapore**
128.	Yuan, et al. (2017) [61]	Not specified	Mixed gender	Mixed Methods	Family Application	Social Interactive Sharing App	Behavioral Pattern Analysis	Moderate technology exposure	Strengthening family ties and promoting communication	Family-focused, emotionally supportive, visually simple interface	Difficulties in adapting to digital interfaces	Improved family interaction and emotional bonding	Further exploring family-specific needs and privacy concerns	**China**
129.	Zhou, et al. (2022) [150]	Not specified	Mixed gender	Mixed Methods	Familiar Interface Language	Mobile Applications	Emotional Design Strategies	Physiological and Psychological Characteristics	Positive emotional feedback, practical visual effects	Difficulty in comprehending digital interfaces	Enhanced emotional connection and ease of use	Further improving interface personalization and accessibility		**China**
130.	Zhou, H., et al. (2024) [60]	N = 140 Age range = 70 +	Mixed gender	Mixed Methods	Chatbot Book Club	Large Language Models	Prompt Engineering	Low digital literacy	Personalized, thought-provoking questions and voice interactivity	Large fonts, user-friendly interface	Difficulty in maintaining cognitive engagement	Improved cognitive function and ongoing engagement	Iterating based on user feedback and exploring more applications	**China**
131.	Zhu, et al. (2022) [151]	N = 15 Age range = 60-75	33% men, 67% women	User Study	To-Do List App	Mobile Applications	UEQ, Interviews	Varied technology use	Memory support, task organization, non-declarative memory assistance	Task completion, simplicity, visual clarity, real-time feedback	Usability issues identified in iteration plan	Enhanced memory retention and task management for older adults	Enhancing perspicuity and reducing complexity	**China**
132.	Zhu, et al. (2022) [19]	N = 200 Age range = 60-80	Mixed gender	Mixed Methods	Interactive Cognitive App	Mobile Applications	Usability Testing, Expert Evaluation	Varied levels of technology use	Daily practice, challenge mode, two-player mode, sharing function	Level-based difficulty, novice teaching, desktop components	Navigating user interface, technical setup difficulties	Improved cognitive training experience, interactive solution for memory improvement	Expanding accessibility features and enhancing real-time feedback mechanisms	**China**

### Key Themes and Findings

#### Design features enhancing accessibility and usability

Across the studies, several design principles were consistently highlighted as critical for improving mobile app usability among older adults:

*Simplified Navigation:* Linear (or simple) navigation paths, clear menu hierarchies, and logical workflows were frequently cited as effective in reducing cognitive load and improving task completion rates.

*Readable Interfaces*: Larger fonts, high-contrast text, and adjustable display settings addressed common age-related visual impairments.

*Touch-Friendly Elements:* Enlarged touch targets and error-tolerant interfaces reduced user frustration associated with fine motor skill declines.

*Voice Interaction and Feedback:* Voice-based controls and audio feedback improved accessibility for users with visual or motor impairments.

#### User-centered and participatory design approaches

Some of the included studies highlighted co-design methods involving older adults. Participatory design processes were linked to higher user satisfaction, as they enabled iterative feedback and customization of app features to address specific needs. For instance, despite perceived challenges, Hakobyan et al. (2014) demonstrated the substantial benefits of directly involving target users in the design process. Their findings further suggest that older adults with impairments can effectively co-design technological solutions tailored to their needs and abilities [8].

Liu and Yu (2024) evaluated the SugarShift (2023) app for diabetes management in older adults, highlighting that its generalized design overlooks elderly users' cognitive and physiological needs. Using a participatory design approach, they identified interface simplicity, logical navigation, and interaction quality as key factors in improving user experience. Their proposed modifications enhanced information processing and reduced cognitive load for older users [9].

#### Challenges in adopting mobile technology

Common barriers to mobile app adoption among older adults include:

*Cognitive Barriers (Cognitive Load & Processing Challenges):* This category includes challenges that are caused by reduced cognitive abilities, mental fatigue, and confusion during interaction. Examples include cognitive decline, forgetfulness, feeling overwhelmed by complex information, and difficulty understanding or navigating apps without a clear and consistent mental model [1, 4, 9–60].

*Technical and experience-based barriers (Technical Literacy & Familiarity Barriers):* "This category covers challenges resulting from a lack of technical skills, limited prior exposure, or general unfamiliarity with using technology. Examples include Unfamiliarity with smartphones, inability to use wearables, need for training, digital divide [1, 16, 23, 24, 43, 44, 50, 51, 53, 57, 61–67].

*Physical and sensory barriers:* This category refers to limitations in interacting with the app due to visual, hearing, or motor impairments. Examples of these barriers include hand tremors, poor vision, inability to accurately touch the screen, and slow Braille reading speed [1, 5, 20, 21, 23, 24, 28, 29, 31, 35, 50, 52, 68–77].

*Psychosocial barriers*: This category includes barriers related to trust, motivation, isolation, social acceptance, or security concerns. Examples include privacy concerns, low trust in technology, social isolation, and interaction stress[25, 47, 55, 73, 78–80].

#### Impact of Age-Friendly Features

Studies consistently reported improvements in usability metrics, such as ease of use, task completion times, and user satisfaction, following the integration of age-friendly design features. For example:

A usability study of a dementia-focused health app (SMART4MD) reported that age-friendly features of the SMART4MD app, such as the thorough introduction, paper-based manual, availability of tablet pens, and customization/personalization options, were found to be crucial in facilitating the adoption and use of the technology by the target user group of older adults with mild cognitive impairment [50].

#### Recommendations for Future App Development

The review identified several areas for improvement and research in mobile app design for older adults:

***Incorporation of Assistive Technologies:*** Integrating AI-driven features such as voice assistants and predictive text could enhance accessibility.

***Cultural and Contextual Sensitivity:*** Designing apps that account for cultural differences and specific user contexts can improve inclusivity.

***Longitudinal Usability Testing:*** Few studies evaluated the long-term impact of age-friendly features on user adoption and sustained engagement, highlighting a critical gap in the literature.

## Discussion

This systematic review explores findings from 132 studies investigating mobile app design features to enhance accessibility, usability, and engagement for older adults. The included studies primarily targeted older adults and explored various design principles, participatory design approaches, and barriers to technology adoption. The results provide valuable insights into optimizing mobile apps for older users and offer actionable recommendations for future development.

### Design features enhancing accessibility and usability

A prominent theme across studies is the importance of simplifying mobile app interfaces to address the unique challenges older adults face, such as age-related declines in vision, hearing, motor skills, and cognitive function, which often create barriers to everyday technologies [81]. Key features like simplified navigation, readable interfaces, touch-friendly elements, and voice interaction have been identified as critical for enhancing usability. Linear navigation paths and logical workflows reduce cognitive load, significantly improving task completion rates [82], while integrating larger fonts, high-contrast text, and adjustable display settings benefits users with visual impairments, aligning with established accessibility principles [5, 69, 83]. Touch-friendly elements, such as enlarged touch targets and error-tolerant interfaces, accommodate motor impairments [82], and voice-based controls with audio feedback enhance accessibility for those with visual or motor limitations [68]. These findings align with universal design principles—such as clear visual contrast, intuitive navigation, and voice-activated controls—which mitigate barriers and improve functional capacity [84, 85]. For example, well-designed assistive technologies, like hearing aids with simplified interfaces or walkers with adjustable grips, reduce fall risks, a leading cause of injury in older adults [85]. Similarly, accessible digital platforms featuring larger fonts and screen reader compatibility enhance social connectivity and access to essential services, reducing isolation [81]. By prioritizing inclusive design, we can create environments and products that meet the diverse needs of aging individuals, fostering autonomy and improving overall well-being.

### User-centered and participatory design approaches

The review highlights the effectiveness of co-design and participatory methods in creating mobile apps and technologies that better meet the needs of older adults. In some of the included studies, participatory design approaches—where older adults were actively involved in the design process—were associated with higher user satisfaction and usability. For example, Putri et al. (2019) demonstrated that incorporating user feedback and iterative design processes led to significant improvements in app functionality and accessibility [86]. hese findings align with broader research advocating for user-centered design, emphasizing the importance of tailoring solutions to older adults' specific needs, preferences, and challenges [36, 74, 87]. Studies have shown that involving older adults in the design of assistive technologies, such as wearable devices or home automation systems, results in more intuitive and functional solutions that align with their daily routines and physical capabilities [88]. Moreover, participatory design fosters a sense of empowerment and ownership among elderly users, enhancing their motivation to adopt and consistently use these innovations [89]. By prioritizing user-centered and participatory design, developers can create more effective and meaningful interventions that improve older adults' usability, independence, and overall well-being [81].

### Challenges in adopting mobile technology

Older adults face significant barriers to adopting mobile technology, primarily due to age-related physical, cognitive, and psychosocial challenges. Cognitive barriers, such as complex interfaces and information overload, are significant obstacles, with many older adults struggling to navigate smartphones and digital interfaces due to low digital literacy [5, 90]. Physical limitations, including fine motor skill impairments and visual challenges, further hinder effective app use, as small touchscreen interfaces, complex menus, and small font sizes exacerbate declines in vision, dexterity, and cognitive processing [81]. Additionally, lack of familiarity with digital devices and fear of making errors contribute to anxiety and resistance toward technology adoption [91]. Research highlights that perceived usefulness and ease of use, as outlined by the Technology Acceptance Model, are critical determinants of technology acceptance among older adults [92]. For instance, older users are more likely to adopt mobile devices if they perceive them as beneficial for staying connected or managing health, However, they often require tailored training and support to overcome initial barriers [93]. Addressing these challenges through user-centered design, simplified interfaces, and accessible training programs is essential for enhancing mobile technology adoption and improving digital inclusion among the elderly.

### Impact of age-friendly features

Integration age-friendly design features consistently improves usability metrics such as ease of use, task completion times, and user satisfaction. For example, apps designed for dementia patients have shown reduced task errors and improved caregiver coordination through simplified interfaces, aligning with research highlighting the positive impact of age-friendly features on app performance and user experience [5, 69, 83]. Adaptive design strategies, such as customizable font sizes and tailored content, further enhance user engagement and reduce abandonment rates, underscoring the importance of personalization in promoting app adoption and sustained use. Beyond digital interfaces, age-friendly design principles—such as ergonomic handles, clear visual contrast, and voice-activated controls—mitigate age-related declines in vision, hearing, and motor skills, making everyday tasks more manageable [94]. For instance, walkers with adjustable grips and non-slip surfaces significantly reduce fall risks, a leading cause of injury among older adults [85]. Age-friendly digital features, including larger fonts, simplified navigation, and screen reader compatibility, also improve accessibility, fostering social connectivity and access to essential services [81]. Similarly, environments designed with age-friendly principles, such as well-lit pathways and accessible public transportation, promote mobility and social participation, reducing isolation and enhancing mental health [95]. By prioritizing age-friendly features, designers and policymakers can create inclusive solutions that support the diverse needs of aging individuals, improving autonomy, independence, and overall well-being.

### Recommendations for future app development

The review identified several key areas for future research and improvement in mobile app design for older adults. First, integrating assistive technologies, such as AI-driven voice assistants and predictive text, could further enhance accessibility for users with cognitive and physical impairments. This aligns with emerging trends in the field, where AI technologies are being increasingly incorporated into health apps to assist users in performing complex tasks more easily. Second, the need for cultural and contextual sensitivity in app design was highlighted. As older adults are highly diverse, mobile apps must consider cultural differences and specific user contexts to ensure inclusivity and relevance. Lastly, longitudinal usability testing was identified as a critical gap in the literature. Few studies evaluated the long-term impact of age-friendly features on user adoption and sustained engagement, suggesting that future research should focus on the enduring effects of these design elements over time.

### Limitations

While this systematic review provides valuable insights into optimizing mobile app design for older adults, several limitations should be acknowledged. First, the heterogeneity of study designs and evaluation metrics made it difficult to conduct a meta-analysis, limiting the ability to compare outcomes across studies quantitatively. Second, the review primarily focused on English-language publications, which may exclude relevant findings from non-English sources. Additionally, variations in the technological proficiency of study participants may have influenced usability outcomes, as older adults with prior digital experience may respond differently to design interventions than those with minimal exposure.This review was not pre-registered in a protocol database such as PROSPERO, which we acknowledge as a limitation. Protocol pre-registration enhances transparency and reduces the risk of bias in systematic reviews. Future research will benefit from this critical step. Additionally, approximately 20.5% of the included studies, primarily descriptive, lacked details on sampling strategies and representativeness, which limited the generalizability of the findings. Furthermore, 9.1% of qualitative studies did not report the influence of researchers, which may introduce bias. Unreported response rates in descriptive and mixed-methods studies further hindered quality assessment. Future research should prioritize detailed reporting of methodological aspects, including sampling, response rates, and researcher influence, and adopt standardized evaluation tools to enhance study quality and comparability.

## Conclusion

This review highlights the importance of designing mobile apps that cater to the unique needs of older adults, emphasizing accessibility, usability, and user engagement. The consistent findings across the included studies underscore the need for simplified, intuitive interfaces, co-design approaches, and adaptive features that address older users' physical and cognitive challenges. However, challenges in mobile technology adoption persist, and future app development must focus on integrating assistive technologies, ensuring cultural sensitivity, and conducting longitudinal usability testing to improve long-term user satisfaction and engagement.

## Supplementary Information

Below is the link to the electronic supplementary material.

Supplementary file1 (DOCX 32 KB)

## Data Availability

No datasets were generated or analysed during the current study.
