# Supplementary material for: Optimizing mobile app design for older adults: systematic review of age-friendly design
Source: Aging Clin Exp Res. 2025 Aug 14;37(1):248. doi: 10.1007/s40520-025-03157-7 (PMC12350549; doi:10.1007/s40520-025-03157-7)
Supplement: Supplementary file 1 — Supplementary file1 (DOCX 32 KB) [file 40520_2025_3157_MOESM1_ESM.docx]

**Table 2- Qualitative Evaluation Using MMAT**

| Study ID | Authors | Study Type | Screening Questions | MMAT Criteria | MMAT Score | Remarks |
| --- | --- | --- | --- | --- | --- | --- |
| 1 | Al-khomsan et al. (2015) | Experimental | Yes, Yes | Randomization: No Blinding: Unclear Outcome Measurement: Yes (Morae) Dropout Rate: Unclear Adherence: Yes | 2/5 | Limited details on randomization and dropout rate. |
| 2 | Ahmed et al. (2014) | Mixed-Methods | Yes, Yes | Qualitative: Yes Quantitative: Yes (Surveys) Integration: Yes Inconsistencies: Unclear Context: Yes | 4/5 | Strong integration; inconsistencies not reported. |
| 3 | Al-Shaher & Abdul-wahed (2020) | Usability Testing (Descriptive) | Yes, Yes | Sampling: Unclear Representativeness: No Measurement: Yes Response Rate: Unclear Statistical Analysis: Unclear | 1/5 | Lack of sampling and representativeness details. |
| 4 | Ali et al. (2024) | Mixed-Methods | Yes, Yes | Qualitative: Yes Quantitative: Yes Integration: Yes Inconsistencies: Yes Context: Yes | 5/5 | Robust design with full integration. |
| 5 | Aljedaani & Alnanih (2023) | Usability Testing (Descriptive) | Yes, Yes | Sampling: Yes Representativeness: Yes Measurement: Yes Response Rate: Unclear Statistical Analysis: Yes | 4/5 | Response rate not reported. |
| 6 | Alkhomsan et al. (2023) | Mixed-Methods | Yes, Yes | Qualitative: Yes Quantitative: Yes Integration: Yes Inconsistencies: Yes Context: Yes | 5/5 | Robust design with cultural considerations. |
| 7 | Alsaqer & Chatterjee (2017) | Intervention (Non-Randomized) | Yes, Yes | Representativeness: Unclear Measurement: Yes Confounders: Unclear Integrity: Yes Statistical Analysis: Yes | 3/5 | Representativeness and confounders unclear. |
| 8 | Alvarez et al. (2017) | Intervention (Non-Randomized) | Yes, Yes | Representativeness: Yes Measurement: Yes Confounders: Unclear Integrity: Yes Statistical Analysis: Yes | 4/5 | Strong representativeness; confounders unclear. |
| 9 | Anastasiadou & Lanitis (2022) | Mixed-Methods | Yes, Yes | Qualitative: Yes Quantitative: Yes Integration: Unclear Inconsistencies: Unclear Context: Yes | 3/5 | Integration unclear. |
| 10 | Ariza-Vega et al. (2024) | Qualitative | Yes, Yes | Data Sources: Yes Analysis: Yes Context: Yes Researcher Influence: Unclear | 3/5 | Researcher influence not reported. |
| 11 | Arruda et al. (2018) | Framework (Mixed-Methods) | Yes, Yes | Qualitative: Yes Quantitative: Yes Integration: Yes Inconsistencies: Unclear Context: Yes | 4/5 | Inconsistencies not reported. |
| 12 | Azam et al. (2021) | User Preference (Mixed-Methods) | Yes, Yes | Qualitative: Yes Quantitative: Yes Integration: Yes Inconsistencies: Yes Context: Yes | 5/5 | Robust design with focus on readability. |
| 13 | Bhattacharyya et al. (2019) | Human-Centered Design (Mixed-Methods) | Yes, Yes | Qualitative: Yes Quantitative: Yes Integration: Yes Inconsistencies: Unclear Context: Yes | 4/5 | Inconsistencies not reported. |
| 14 | Bhayana et al. (2020) | Application (Mixed-Methods) | Yes, Yes | Qualitative: Yes Quantitative: Yes Integration: Yes Inconsistencies: Yes Context: Yes | 5/5 | Strong integration with social interaction focus. |
| 15 | Boccardi et al. (2022) | Mixed-Methods | Yes, Yes | Qualitative: Yes Quantitative: Yes Integration: Yes Inconsistencies: Yes Context: Yes | 5/5 | Robust design for wheelchair users. |
| 16 | Calderón-Gómez et al. (2020) | Observational (Non-Randomized) | Yes, Yes | Representativeness: Yes Measurement: Yes Confounders: Yes Integrity: Yes Statistical Analysis: Yes | 5/5 | Robust design with machine learning analysis. |
| 17 | Calyam et al. (2017) | Living Lab (Mixed-Methods) | Yes, Yes | Qualitative: Yes Quantitative: Yes Integration: Yes Inconsistencies: Unclear Context: Yes | 4/5 | Inconsistencies not reported. |
| 18 | Carrisa et al. (2022) | Case Study (Mixed-Methods) | Yes, Yes | Qualitative: Yes Quantitative: Yes Integration: Yes Inconsistencies: Yes Context: Yes | 5/5 | Robust design with automated testing. |
| 19 | Casciaro et al. (2020) | Smart Device (Mixed-Methods) | Yes, Yes | Qualitative: Yes Quantitative: Yes Integration: Yes Inconsistencies: Yes Context: Yes | 5/5 | Robust design with medication adherence focus. |
| 20 | Chang et al. (2022) | Mobile App (Mixed-Methods) | Yes, Yes | Qualitative: Yes Quantitative: Yes Integration: Yes Inconsistencies: Unclear Context: Yes | 4/5 | Inconsistencies not reported. |
| 21 | Chao et al. (2024) | Case Study (Mixed-Methods) | Yes, Yes | Qualitative: Yes Quantitative: Yes Integration: Yes Inconsistencies: Unclear Context: Yes | 4/5 | Inconsistencies not reported. |
| 22 | Charissis et al. (2024) | XR Design (Mixed-Methods) | Yes, Yes | Qualitative: Yes Quantitative: Yes Integration: Yes Inconsistencies: Yes Context: Yes | 5/5 | Robust design with XR technology. |
| 23 | Chen & Gao (2021) | Unspecified | Yes, Yes | Qualitative: No Quantitative: No Integration: No Inconsistencies: No Context: Yes | 1/5 | Incomplete methodological details. |
| 24 | Chen et al. (2022) | Empirical Investigation (Descriptive) | Yes, Yes | Sampling: No Representativeness: No Measurement: Yes Response Rate: Unclear Statistical Analysis: Yes | 2/5 | Lack of sampling and representativeness. |
| 25 | Chien (2024) | Usability Study (Descriptive) | Yes, Yes | Sampling: Yes Representativeness: Yes Measurement: Yes Response Rate: Unclear Statistical Analysis: Yes | 4/5 | Response rate not reported. |
| 26 | Chirayus & Nanthaamornphong (2020) | Preliminary Study (Descriptive) | Yes, Yes | Sampling: No Representativeness: No Measurement: Yes Response Rate: Unclear Statistical Analysis: Yes | 2/5 | Lack of sampling and representativeness. |
| 27 | Choi et al. (2021) | Descriptive Exploratory (Qualitative) | Yes, Yes | Data Sources: Yes Analysis: Yes Context: Yes Researcher Influence: Unclear | 3/5 | Researcher influence not reported. |
| 28 | Cordasco et al. (2014) | Usability Evaluation (Descriptive) | Yes, Yes | Sampling: No Representativeness: No Measurement: Yes Response Rate: Unclear Statistical Analysis: Yes | 2/5 | Lack of sampling and representativeness. |
| 29 | Cornet et al. (2020) | Qualitative | Yes, Yes | Data Sources: Yes Analysis: Yes Context: Yes Researcher Influence: Unclear | 3/5 | Researcher influence not reported. |
| 30 | Correia et al. (2024) | Usability Study (Descriptive) | Yes, Yes | Sampling: No Representativeness: No Measurement: Yes Response Rate: Unclear Statistical Analysis: Yes | 2/5 | Lack of sampling and representativeness. |
| 31 | Craioveanu & Marcu (2023) | Usability Study (Descriptive) | Yes, Yes | Sampling: No Representativeness: No Measurement: Yes Response Rate: Unclear Statistical Analysis: Yes | 2/5 | Lack of sampling and representativeness. |
| 32 | Cristiano et al. (2018) | Validation Study (Descriptive) | Yes, Yes | Sampling: Yes Representativeness: Yes Measurement: Yes Response Rate: Unclear Statistical Analysis: Yes | 4/5 | Response rate not reported. |
| 33 | Curiel et al. (2014) | Comparative Study (Descriptive) | Yes, Yes | Sampling: No Representativeness: No Measurement: Yes Response Rate: Unclear Statistical Analysis: Yes | 2/5 | Lack of sampling and representativeness. |
| 34 | Czuber et al. (2024) | Mixed-Methods | Yes, Yes | Qualitative: Yes Quantitative: Yes Integration: Yes Inconsistencies: Yes Context: Yes | 5/5 | Robust design with focus groups. |
| 35 | Darmawan et al. (2023) | Mixed-Methods | Yes, Yes | Qualitative: Yes Quantitative: Yes Integration: Yes Inconsistencies: Yes Context: Yes | 5/5 | Robust design with AMOS analysis. |
| 36 | De Melo et al. (2016) | Case Study (Mixed-Methods) | Yes, Yes | Qualitative: Yes Quantitative: Yes Integration: Yes Inconsistencies: Yes Context: Yes | 5/5 | Robust user-centered design. |
| 37 | Diewald et al. (2015) | User-Centered Design (Mixed-Methods) | Yes, Yes | Qualitative: Yes Quantitative: Yes Integration: Yes Inconsistencies: Unclear Context: Yes | 4/5 | Inconsistencies not reported. |
| 38 | Doménech et al. (2023) | Observational (Non-Randomized) | Yes, Yes | Representativeness: Yes Measurement: Yes Confounders: Yes Integrity: Yes Statistical Analysis: Yes | 5/5 | Robust design with VAS and PANAS analysis. |
| 39 | Dos Santos et al. (2016) | Qualitative | Yes, Yes | Data Sources: Yes Analysis: Yes Context: Yes Researcher Influence: Unclear | 3/5 | Researcher influence not reported. |
| 40 | Držanič et al. (2019) | Usability Study (Descriptive) | Yes, Yes | Sampling: Yes Representativeness: Yes Measurement: Yes Response Rate: Unclear Statistical Analysis: Yes | 4/5 | Response rate not reported. |
| 41 | Dworschak et al. (2024) | Development Study (Mixed-Methods) | Yes, Yes | Qualitative: Yes Quantitative: Yes Integration: Yes Inconsistencies: Yes Context: Yes | 5/5 | Robust design with CBT. |
| 42 | Eicher & Ursprung (2024) | Development Study (Mixed-Methods) | Yes, Yes | Qualitative: Yes Quantitative: Yes Integration: Yes Inconsistencies: Unclear Context: Yes | 4/5 | Inconsistencies not reported. |
| 43 | Eun et al. (2022) | Pilot Study (Mixed-Methods) | Yes, Yes | Qualitative: Yes Quantitative: Yes Integration: Yes Inconsistencies: Yes Context: Yes | 5/5 | Robust design with cognitive gaming. |
| 44 | Ferre et al. (2017) | Iterative Development (Descriptive) | Yes, Yes | Sampling: Yes Representativeness: Yes Measurement: Yes Response Rate: Unclear Statistical Analysis: Yes | 4/5 | Response rate not reported. |
| 45 | Forbes et al. (2024) | Qualitative | Yes, Yes | Data Sources: Yes Analysis: Yes Context: Yes Researcher Influence: Unclear | 3/5 | Researcher influence not reported. |
| 46 | Frechette et al. (2022) | Observational (Descriptive) | Yes, Yes | Sampling: Yes Representativeness: Yes Measurement: Yes Response Rate: Unclear Statistical Analysis: Yes | 4/5 | Response rate not reported. |
| 47 | Frogren et al. (2018) | Feasibility Study (Mixed-Methods) | Yes, Yes | Qualitative: Yes Quantitative: Yes Integration: Yes Inconsistencies: Yes Context: Yes | 5/5 | Robust design with task-based testing. |
| 48 | Fuglerud et al. (2018) | Design and Testing (Qualitative) | Yes, Yes | Data Sources: Yes Analysis: Yes Context: Yes Researcher Influence: Unclear | 3/5 | Researcher influence not reported. |
| 49 | García-Crespo et al. (2020) | Usability Study (Descriptive) | Yes, Yes | Sampling: Yes Representativeness: Yes Measurement: Yes Response Rate: Unclear Statistical Analysis: Yes | 4/5 | Response rate not reported. |
| 50 | Giraldo et al. (2015) | Case Study (Mixed-Methods) | Yes, Yes | Qualitative: Yes Quantitative: Yes Integration: Yes Inconsistencies: Yes Context: Yes | 5/5 | Robust design with elderly care focus. |
| 51 | Griffin et al. (2019) | Case Study (Mixed-Methods) | Yes, Yes | Qualitative: Yes Quantitative: Yes Integration: Yes Inconsistencies: Yes Context: Yes | 5/5 | Robust design with virtual human technology. |
| 52 | Ha et al. (2023) | Usability Study (Descriptive) | Yes, Yes | Sampling: Yes Representativeness: Yes Measurement: Yes Response Rate: Unclear Statistical Analysis: Yes | 4/5 | Response rate not reported. |
| 53 | Hakobyan et al. (2014) | Participatory Design (Mixed-Methods) | Yes, Yes | Qualitative: Yes Quantitative: Yes Integration: Yes Inconsistencies: Unclear Context: Yes | 4/5 | Inconsistencies not reported. |
| 54 | Hammour et al. (2024) | Transfer Learning (Descriptive) | Yes, Yes | Sampling: Yes Representativeness: Yes Measurement: Yes Response Rate: Unclear Statistical Analysis: Yes | 4/5 | Response rate not reported. |
| 55 | Hanghøj et al. (2020) | Co-creation (Mixed-Methods) | Yes, Yes | Qualitative: Yes Quantitative: Yes Integration: Yes Inconsistencies: Yes Context: Yes | 5/5 | Robust design with thematic analysis. |
| 56 | Happe et al. (2022) | Usability Study (Descriptive) | Yes, Yes | Sampling: Yes Representativeness: Yes Measurement: Yes Response Rate: Unclear Statistical Analysis: Yes | 4/5 | Response rate not reported. |
| 57 | Harte et al. (2018) | Usability Study (Descriptive) | Yes, Yes | Sampling: Yes Representativeness: Yes Measurement: Yes Response Rate: Unclear Statistical Analysis: Yes | 4/5 | Response rate not reported. |
| 58 | Harte et al. (2017) | Human-Centered Design (Descriptive) | Yes, Yes | Sampling: No Representativeness: No Measurement: Yes Response Rate: Unclear Statistical Analysis: Yes | 2/5 | Lack of sampling and representativeness. |
| 59 | Heiney et al. (2020) | Feasibility Study (Mixed-Methods) | Yes, Yes | Qualitative: Yes Quantitative: Yes Integration: Yes Inconsistencies: Yes Context: Yes | 5/5 | Robust design with HQOL14 and SCHFI tools. |
| 60 | Huang et al. (2024) | Mixed-Methods | Yes, Yes | Qualitative: Yes Quantitative: Yes Integration: Yes Inconsistencies: Yes Context: Yes | 5/5 | Robust design with community collaboration. |
| 61 | Huwa et al. (2023) | Usability & Acceptability (Mixed-Methods) | Yes, Yes | Qualitative: Yes Quantitative: Yes Integration: Yes Inconsistencies: Yes Context: Yes | 5/5 | Robust design with high satisfaction (95%). |
| 62 | Iqbal et al. (2020) | Proof of Concept (Descriptive) | Yes, Yes | Sampling: No Representativeness: No Measurement: Yes Response Rate: Unclear Statistical Analysis: Yes | 2/5 | Lack of sampling and representativeness. |
| 63 | Ismail et al. (2021) | Usability Testing (Descriptive) | Yes, Yes | Sampling: Yes Representativeness: Yes Measurement: Yes Response Rate: Unclear Statistical Analysis: Yes | 4/5 | Response rate not reported. |
| 64 | Jakkaew & Hongthong (2017) | Usability Study (Descriptive) | Yes, Yes | Sampling: No Representativeness: No Measurement: Yes Response Rate: Unclear Statistical Analysis: Yes | 2/5 | Lack of sampling and representativeness. |
| 65 | Jiang et al. (2024) | Factor Analysis (Descriptive) | Yes, Yes | Sampling: No Representativeness: No Measurement: Yes Response Rate: Unclear Statistical Analysis: Yes | 2/5 | Lack of sampling and representativeness. |
| 66 | Kalyani et al. (2022) | Development Study (Descriptive) | Yes, Yes | Sampling: No Representativeness: No Measurement: Yes Response Rate: Unclear Statistical Analysis: Yes | 2/5 | Lack of sampling and representativeness. |
| 67 | Kangeswaran et al. (2021) | Assistive Technology (Descriptive) | Yes, Yes | Sampling: No Representativeness: No Measurement: Yes Response Rate: Unclear Statistical Analysis: Yes | 2/5 | Lack of sampling and representativeness. |
| 68 | Kim et al. (2020) | Development Study (Mixed-Methods) | Yes, Yes | Qualitative: Yes Quantitative: Yes Integration: Yes Inconsistencies: Yes Context: Yes | 5/5 | Robust design with social interaction focus. |
| 69 | Kim et al. (2024) | Usability Study (Descriptive) | Yes, Yes | Sampling: Yes Representativeness: Yes Measurement: Yes Response Rate: Unclear Statistical Analysis: Yes | 4/5 | Response rate not reported. |
| 70 | Klimova & Sanda (2021) | Pilot Study (Descriptive) | Yes, Yes | Sampling: Yes Representativeness: Yes Measurement: Yes Response Rate: Unclear Statistical Analysis: Yes | 4/5 | Response rate not reported. |
| 71 | Anon (2021) | Mixed-Methods | Yes, Yes | Qualitative: Yes Quantitative: Yes Integration: Yes Inconsistencies: Yes Context: Yes | 5/5 | Robust design with IGUAN framework. |
| 72 | Kunaratana-Angkul et al. (2020) | Usability Evaluation (Descriptive) | Yes, Yes | Sampling: No Representativeness: No Measurement: Yes Response Rate: Unclear Statistical Analysis: Yes | 2/5 | Lack of sampling and representativeness. |
| 73 | Liu et al. (2019) | Randomized Controlled Trial | Yes, Yes | Randomization: Yes Blinding: Unclear Outcome Measurement: Yes Dropout Rate: Unclear Adherence: Yes | 3/5 | Blinding and dropout rate not reported. |
| 74 | Liu & Yu (2024) | Participatory Design (Mixed-Methods) | Yes, Yes | Qualitative: Yes Quantitative: Yes Integration: Yes Inconsistencies: Yes Context: Yes | 5/5 | Robust design with diabetes focus. |
| 75 | Margaritini et al. (2022) | Study Protocol (Qualitative) | Yes, Yes | Data Sources: Yes Analysis: Yes Context: Yes Researcher Influence: Unclear | 3/5 | Researcher influence not reported. |
| 76 | Mehra et al. (2019) | Usability Evaluation (Descriptive) | Yes, Yes | Sampling: Yes Representativeness: Yes Measurement: Yes Response Rate: Unclear Statistical Analysis: Yes | 4/5 | Response rate not reported. |
| 77 | Merilampi et al. (2017) | Participatory Design (Mixed-Methods) | Yes, Yes | Qualitative: Yes Quantitative: Yes Integration: Yes Inconsistencies: Yes Context: Yes | 5/5 | Robust design with cultural content. |
| 78 | Molnar (2015) | Usability Evaluation (Descriptive) | Yes, Yes | Sampling: Yes Representativeness: Yes Measurement: Yes Response Rate: Unclear Statistical Analysis: Yes | 4/5 | Response rate not reported. |
| 79 | Mondellini et al. (2018) | Pilot Study (Descriptive) | Yes, Yes | Sampling: No Representativeness: No Measurement: Yes Response Rate: Unclear Statistical Analysis: Yes | 2/5 | Lack of sampling and representativeness. |
| 80 | Morris et al. (2024) | Case Study (Qualitative) | Yes, Yes | Data Sources: Yes Analysis: Yes Context: Yes Researcher Influence: Unclear | 3/5 | Researcher influence not reported. |
| 81 | Nair et al. (2022) | Usability Evaluation (Descriptive) | Yes, Yes | Sampling: Yes Representativeness: Yes Measurement: Yes Response Rate: Unclear Statistical Analysis: Yes | 4/5 | Response rate not reported. |
| 82 | Anon (2022) | Heuristic Evaluation (Descriptive) | Yes, Yes | Sampling: No Representativeness: No Measurement: Yes Response Rate: Unclear Statistical Analysis: Yes | 2/5 | Lack of sampling and representativeness. |
| 83 | Park et al. (2024) | Development & Usability (Mixed-Methods) | Yes, Yes | Qualitative: Yes Quantitative: Yes Integration: Yes Inconsistencies: Yes Context: Yes | 5/5 | Robust design with wearable integration. |
| 84 | Petrovčič et al. (2019) | Developmental/Experimental (Descriptive) | Yes, Yes | Sampling: Yes Representativeness: Yes Measurement: Yes Response Rate: Unclear Statistical Analysis: Yes | 4/5 | Response rate not reported. |
| 85 | Pinto & Marques (2017) | Proof of Concept (Descriptive) | Yes, Yes | Sampling: Yes Representativeness: Yes Measurement: Yes Response Rate: Unclear Statistical Analysis: Yes | 4/5 | Response rate not reported. |
| 86 | Porcel Gálvez et al. (2024) | Platform Development (Descriptive) | Yes, Yes | Sampling: No Representativeness: No Measurement: Yes Response Rate: Unclear Statistical Analysis: Yes | 2/5 | Lack of sampling and representativeness. |
| 87 | Puebla et al. (2022) | Design-Thinking (Mixed-Methods) | Yes, Yes | Qualitative: Yes Quantitative: Yes Integration: Yes Inconsistencies: Yes Context: Yes | 5/5 | Robust design with collaborative learning. |
| 88 | Putri et al. (2019) | User-Centered Design (Descriptive) | Yes, Yes | Sampling: No Representativeness: No Measurement: Yes Response Rate: Unclear Statistical Analysis: Yes | 2/5 | Lack of sampling and representativeness. |
| 89 | Quesada et al. (2024) | Multi-Case Study (Mixed-Methods) | Yes, Yes | Qualitative: Yes Quantitative: Yes Integration: Yes Inconsistencies: Yes Context: Yes | 5/5 | Robust design with intelligent assistants. |
| 90 | Quintana et al. (2020) | Feasibility & Usability (Mixed-Methods) | Yes, Yes | Qualitative: Yes Quantitative: Yes Integration: Yes Inconsistencies: Yes Context: Yes | 5/5 | Robust design with high satisfaction (81%). |
| 91 | Radhakrishnan et al. (2016) | Feasibility & Usability (Mixed-Methods) | Yes, Yes | Qualitative: Yes Quantitative: Yes Integration: Yes Inconsistencies: Yes Context: Yes | 5/5 | Robust design with gamification. |
| 92 | Raghavendra et al. (2024) | Usability Testing (Descriptive) | Yes, Yes | Sampling: Yes Representativeness: Yes Measurement: Yes Response Rate: Unclear Statistical Analysis: Yes | 4/5 | Response rate not reported. |
| 93 | Ran et al. (2024) | Empirical Investigation (Descriptive) | Yes, Yes | Sampling: No Representativeness: No Measurement: Yes Response Rate: Unclear Statistical Analysis: Yes | 2/5 | Lack of sampling and representativeness. |
| 94 | Rangel et al. (2019) | SAR Development (Descriptive) | Yes, Yes | Sampling: No Representativeness: No Measurement: Yes Response Rate: Unclear Statistical Analysis: Yes | 2/5 | Lack of sampling and representativeness. |
| 95 | Rasche et al. (2015) | Empirical Study (Descriptive) | Yes, Yes | Sampling: Yes Representativeness: Yes Measurement: Yes Response Rate: Unclear Statistical Analysis: Yes | 4/5 | Response rate not reported. |
| 96 | Rath & Chandna (2021) | Conceptual Study (Descriptive) | Yes, Yes | Sampling: No Representativeness: No Measurement: Yes Response Rate: Unclear Statistical Analysis: Yes | 2/5 | Lack of sampling and representativeness. |
| 97 | Reading Turchioe et al. (2020) | Cross-Sectional Feasibility (Mixed-Methods) | Yes, Yes | Qualitative: Yes Quantitative: Yes Integration: Yes Inconsistencies: Yes Context: Yes | 5/5 | Robust design with high usability. |
| 98 | Riaz et al. (2021) | Qualitative | Yes, Yes | Data Sources: Yes Analysis: Yes Context: Yes Researcher Influence: Unclear | 3/5 | Researcher influence not reported. |
| 99 | Rodríguez-Dueñas et al. (2021) | Pilot Study (Descriptive) | Yes, Yes | Sampling: No Representativeness: No Measurement: Yes Response Rate: Unclear Statistical Analysis: Yes | 2/5 | Lack of sampling and representativeness. |
| 100 | Rosman et al. (2023) | Usability Testing (Descriptive) | Yes, Yes | Sampling: No Representativeness: No Measurement: Yes Response Rate: Unclear Statistical Analysis: Yes | 2/5 | Lack of sampling and representativeness. |
| 101 | Saari & Hynninen (2021) | Usability Testing (Descriptive) | Yes, Yes | Sampling: No Representativeness: No Measurement: Yes Response Rate: Unclear Statistical Analysis: Yes | 2/5 | Lack of sampling and representativeness. |
| 102 | Schaaf et al. (2024) | Qualitative | Yes, Yes | Data Sources: Yes Analysis: Yes Context: Yes Researcher Influence: Unclear | 3/5 | Researcher influence not reported. |
| 103 | Shi-Ning & Yuanqing (2022) | Qualitative | Yes, Yes | Data Sources: Yes Analysis: Yes Context: Yes Researcher Influence: Unclear | 3/5 | Researcher influence not reported. |
| 104 | Shore et al. (2020) | Pilot Study (Descriptive) | Yes, Yes | Sampling: Yes Representativeness: Yes Measurement: Yes Response Rate: Unclear Statistical Analysis: Yes | 4/5 | Response rate not reported. |
| 105 | Siangpipop et al. (2023) | User-Centered Design (Descriptive) | Yes, Yes | Sampling: Yes Representativeness: Yes Measurement: Yes Response Rate: Unclear Statistical Analysis: Yes | 4/5 | Response rate not reported. |
| 106 | Sien et al. (2024) | User-Centered Design (Mixed-Methods) | Yes, Yes | Qualitative: Yes Quantitative: Yes Integration: Yes Inconsistencies: Yes Context: Yes | 5/5 | Robust design with thematic analysis. |
| 107 | Sifan et al. (2021) | User-Centered Design (Descriptive) | Yes, Yes | Sampling: No Representativeness: No Measurement: Yes Response Rate: Unclear Statistical Analysis: Yes | 2/5 | Lack of sampling and representativeness. |
| 108 | Silva et al. (2015) | Heuristic Evaluation (Descriptive) | Yes, Yes | Sampling: Yes Representativeness: Yes Measurement: Yes Response Rate: Unclear Statistical Analysis: Yes | 4/5 | Response rate not reported. |
| 109 | Sinabell & Ammenwerth (2024) | Triangulation Study (Mixed-Methods) | Yes, Yes | Qualitative: Yes Quantitative: Yes Integration: Yes Inconsistencies: Yes Context: Yes | 5/5 | Robust design with agile methods. |
| 110 | Smith-Turchyn et al. (2017) | Usability Study (Mixed-Methods) | Yes, Yes | Qualitative: Yes Quantitative: Yes Integration: Yes Inconsistencies: Yes Context: Yes | 5/5 | Robust design with cognitive interviewing. |
| 111 | Sobnath et al. (2016) | Usability Study (Descriptive) | Yes, Yes | Sampling: No Representativeness: No Measurement: Yes Response Rate: Unclear Statistical Analysis: Yes | 2/5 | Lack of sampling and representativeness. |
| 112 | Sobrinho et al. (2024) | Usability Study (Mixed-Methods) | Yes, Yes | Qualitative: Yes Quantitative: Yes Integration: Yes Inconsistencies: Yes Context: Yes | 5/5 | Robust design with SAM and SUS tools. |
| 113 | Son et al. (2023) | Usability Study (Mixed-Methods) | Yes, Yes | Qualitative: Yes Quantitative: Yes Integration: Yes Inconsistencies: Yes Context: Yes | 5/5 | Robust design with online screening. |
| 114 | Son & Kim (2023) | Mixed-Methods | Yes, Yes | Qualitative: Yes Quantitative: Yes Integration: Yes Inconsistencies: Yes Context: Yes | 5/5 | Robust design with eHealth literacy focus. |
| 115 | Teh et al. (2024) | Qualitative | Yes, Yes | Data Sources: Yes Analysis: Yes Context: Yes Researcher Influence: Unclear | 3/5 | Researcher influence not reported. |
| 116 | Tonga et al. (2021) | Mixed-Methods | Yes, Yes | Qualitative: Yes Quantitative: Yes Integration: Yes Inconsistencies: Yes Context: Yes | 5/5 | Robust design with USE and SUS tools. |
| 117 | Toyota et al. (2014) | Usability Testing (Descriptive) | Yes, Yes | Sampling: No Representativeness: No Measurement: Yes Response Rate: Unclear Statistical Analysis: Yes | 2/5 | Lack of sampling and representativeness. |
| 118 | Tran-Nguyen et al. (2022) | User-Centered Design (Mixed-Methods) | Yes, Yes | Qualitative: Yes Quantitative: Yes Integration: Yes Inconsistencies: Yes Context: Yes | 5/5 | Robust design with workshops. |
| 119 | Tu et al. (2023) | Usability Testing (Descriptive) | Yes, Yes | Sampling: No Representativeness: No Measurement: Yes Response Rate: Unclear Statistical Analysis: Yes | 2/5 | Lack of sampling and representativeness. |
| 120 | Ubam et al. (2021) | Usability Testing (Descriptive) | Yes, Yes | Sampling: Yes Representativeness: Yes Measurement: Yes Response Rate: Unclear Statistical Analysis: Yes | 4/5 | Response rate not reported. |
| 121 | Wahab et al. (2021) | Quantitative Survey (Descriptive) | Yes, Yes | Sampling: No Representativeness: No Measurement: Yes Response Rate: Unclear Statistical Analysis: Yes | 2/5 | Lack of sampling and representativeness. |
| 122 | Wang et al. (2024) | Randomized Crossover | Yes, Yes | Randomization: Yes Blinding: Unclear Outcome Measurement: Yes Dropout Rate: Unclear Adherence: Yes | 3/5 | Blinding and dropout rate not reported. |
| 123 | Wang et al. (2022) | Usability Study (Descriptive) | Yes, Yes | Sampling: No Representativeness: No Measurement: Yes Response Rate: Unclear Statistical Analysis: Yes | 2/5 | Lack of sampling and representativeness. |
| 124 | Xiong et al. (2019) | Qualitative/Quantitative (Mixed-Methods) | Yes, Yes | Qualitative: Yes Quantitative: Yes Integration: Yes Inconsistencies: Yes Context: Yes | 5/5 | Robust design with ARCS motivation model. |
| 125 | Xu & Wen (2024) | Mixed-Methods | Yes, Yes | Qualitative: Yes Quantitative: Yes Integration: Yes Inconsistencies: Yes Context: Yes | 5/5 | Robust design with deep learning. |
| 126 | Yee et al. (2024) | Mixed-Methods | Yes, Yes | Qualitative: Yes Quantitative: Yes Integration: Yes Inconsistencies: Yes Context: Yes | 5/5 | Robust design with personalized AI. |
| 127 | Yeo et al. (2015) | Mixed-Methods | Yes, Yes | Qualitative: Yes Quantitative: Yes Integration: Yes Inconsistencies: Yes Context: Yes | 5/5 | Robust design with speech-to-text. |
| 128 | Yuan et al. (2017) | Mixed-Methods | Yes, Yes | Qualitative: Yes Quantitative: Yes Integration: Yes Inconsistencies: Yes Context: Yes | 5/5 | Robust design with family focus. |
| 129 | Zhou et al. (2022) | Mixed-Methods | Yes, Yes | Qualitative: Yes Quantitative: Yes Integration: Yes Inconsistencies: Yes Context: Yes | 5/5 | Robust design with emotional design strategies. |
| 130 | Zhou et al. (2024) | Mixed-Methods | Yes, Yes | Qualitative: Yes Quantitative: Yes Integration: Yes Inconsistencies: Yes Context: Yes | 5/5 | Robust design with large language models. |
| 131 | Zhu et al. (2022) | User Study (Mixed-Methods) | Yes, Yes | Qualitative: Yes Quantitative: Yes Integration: Yes Inconsistencies: Yes Context: Yes | 5/5 | Robust design with UEQ tool. |
| 132 | Zhu et al. (2022) | Mixed-Methods | Yes, Yes | Qualitative: Yes Quantitative: Yes Integration: Yes Inconsistencies: Yes Context: Yes | 5/5 | Robust design with interactive cognitive app. |
